# *Streptomyces* sp. strain TOR3209: a rhizosphere bacterium promoting growth of tomato by affecting the rhizosphere microbial community

**DOI:** 10.1038/s41598-020-76887-5

**Published:** 2020-11-18

**Authors:** Dong Hu, Shuhong Li, Ying Li, Jieli Peng, Xiaoyan Wei, Jia Ma, Cuimian Zhang, Nan Jia, Entao Wang, Zhanwu Wang

**Affiliations:** 1 Key Laboratory of Plants Genetic Engineering Center, Institute of Genetics and Physiology (Hebei Agricultural Products Quality and Safety Research Center), Hebei Academy of Agriculture and Forestry Sciences, Shijiazhuang, Hebei 050000 People’s Republic of China; 2 Departamento de Microbiología, Escuela Nacional de Ciencias Biológicas, Instituto Politécnico Nacional, C.P. 11340 Mexico City, Mexico

**Keywords:** Microbiology, Bacteria, Microbial communities

## Abstract

Aiming at revealing the possible mechanism of its growth promoting effect on tomato, the correlations among *Streptomyces* sp. TOR3209 inoculation, rhizobacteriome, and tomato growth/production traits were investigated in this study. By analyses of Illumina sequencing and plate coating, differences in rhizosphere microbial communities were found in different growth stages and distinct inoculation treatments. The plant biomass/fruit yields and relative abundances of families *Flavobacteriaceae*, *Sphingobacteriaceae*, *Polyangiaceae* and *Enterobacteriaceae* in treatments T (tomato inoculated with TOR3209) and TF (tomato inoculated with TOR3209 + organic fertilizer) were higher than that in the controls (CK and CK+ organic fertilizer), respectively. The analysis of Metastats and LEfSe revealed that the genera *Flavobacterium* and *Sorangium* in seedling stage, *Klebsiella* in flowering stage, *Collimonas* in early fruit setting stage, and genera *Micrococcaceae*, *Pontibacte* and *Adhaeribacter* in late fruit setting stage were the most representative rhizobacteria that positively responded to TOR3209 inoculation. By cultivation method, five bacterial strains positively correlated to TOR3209 inoculation were isolated from rhizosphere and root endosphere, which were identified as tomato growth promoters affiliated to *Enterobacter* sp., *Arthrobacter* sp., *Bacillus subtilis, Rhizobium* sp. and *Bacillus velezensis*. In pot experiment, TOR3209 and *B. velezensis* WSW007 showed joint promotion to tomato production, while the abundance of inoculated TOR3209 was dramatically decreased in rhizosphere along the growth of tomato. Conclusively, TOR3209 might promote the tomato production via changing of microbial community in rhizosphere. These findings provide a better understanding of the interactions among PGPR in plant promotion.

## Introduction

Rhizosphere refers to the narrow region of soil in the vicinity of plant roots, where the physicochemical traits of soil and the microbiome are quite different from those in bulk soil, because the influence of root secretions and metabolism. The diverse and abundant (up to 10^9^ CFU/g) microbes in rhizosphere can directly or indirectly affect the plant growth and productivity by their metabolic activities^1^. The composition of rhizosphere microbiome is different for distinct crops and it could change accordingly to meet the needs of plant growth at different growth stages^2–4^. In rhizosphere, the bacteria so called plant growth promoting rhizobacteria (PGPR)^5^ could promote plant growth by various mechanisms, such as inhibiting phytopathogens, improving plant nutrient supply, producing phytohormones, and modifying physicochemical properties of soil^6^. On the other hand, PGPR also can affect the composition of microbial communities in the rhizosphere (rhizomicrobiome) of associated plants, which in turn affect the health of the entire soil microbiome^7,8^ and affect the growth of plants. Zhang et al.^9^ identified a consortium of three PGPR strains (*Bacillus cereus* AR156, *Bacillus subtilis* SM21, and *Serratia* sp. XY21) as a promising and biocontrol agent. These strains can change the soil bacterial community and enhancing the disease resistance of plants. Some other studies have shown that PGPR can improve plant resistance and promote plant growth by changing soil microbial community^10,11^.

Although many studies have demonstrated that PGPR play a key role in affecting the biological characteristics of plants through changes in composition and structure of rhizobiome, most of the studies have focused on only one time point and one influencing factors, and the regulation of rhizomicrobiome by a certain microorganism in the entire life cycle of plant growth has been rarely studied^7^. Currently, non-cultivation methods with high-throughput sequencing technology have been widely applied in studies on rhizomicrobiome that overcome the limits of microbial culture and isolation technology. These non-cultivation methods have greatly improved our understanding about the composition and function of microbiome in different environments, and provided a more direct way to detect the microbes, especially those difficult to isolate and cultivate. Anyway, the cultivation methods are still needed to clarify the biological mechanism of the interaction among the microbes and microbes-plants^12^. Therefore, the combination of culture-independent and cultivation methods can explain the growth promoting mechanism of PGPR more comprehensively.

Previously, *Streptomyces* has been reported as one of the PGPR^13^, and *Streptomyces* sp. TOR3209 was isolated from rhizosphere of tomato as a PGP strain in our previous study^14^. The inoculation of this strain could increase the disease resistance and promote growth of several plants, including pear trees, tomatoes and golden silk jujube^15,16^. However, the mechanism of these effects is unknown. Our previous study found that the growth promoting effect of TOR3209 under aseptic condition was not obvious. Therefore, we hypothesize that TOR3209 might benefit the plants by affecting the rhizosmicrobiome, such as stimulating the other PGPR and inhibiting the phytopathogenic microorganisms. In this study, we comparatively analyzed the bacterial community structure in rhizosphere of tomato with/without TOR3209 inoculation in different growth stages using the Illumina Hiseq platform, and tried to isolate the bacteria up-regulated in rhizosphere by TOR3209. The aims of this study were (1) to evaluate whether *Streptomyces* sp*.* TOR3209 could change the rhizobiome and in turn to achieve the growth promotion effects; and (2) to discover the PGP effects of microbial species that responded to the TOR3209 inoculation for further explaining its PGP mechanism.

## Materials and methods

### Pot experiment designation

The strain *Stremptomyces* sp. TOR3209 was originally isolated from rhizosphere of tomato^14^ and its inoculation could increase the growth of tomato and other plants^15,16^. To performed the present study, tomato plants with/without inoculation of this strain was cultured for obtaining the rhizosphere samples, and for confirming its PGP ability as well. Seeds of the tomato cultivar Kaixuan were purchased from Shenyang Huihao Seed Company. The substrate for culturing the tomato seedlings was bought from the Shijiazhuang Nongyou Biotech Co. Ltd. (see Suppl. Table S1 for physicochemical properties). Seeds were grown in the 32-well seedling tray (54 cm × 29 cm × 5 cm, 1 seed per well) with tomato seedling substrate in a greenhouse at 25 ± 3 °C and relative humidity of 75% with a 12 h–12 h light–dark cycle^17^. Seedlings with similar height (15 ± 3 cm) were transplanted into pots (diameter 20 cm) filled with 3.2 kg of different substrate per pot according to the treatments: (i) natural loam soil obtained from Hebei Academy of Agriculture and Forestry Sciences where corn was once planted (see Suppl. Table S1 for physicochemical properties) (CK); (ii) natural loam soil + TOR3209 at the dose of 10^7^ CFU/g (T); (iii) natural loam soil + 10% (w/w) organic fertilizer (F) purchased from Shijiazhuang Golden Sun Microbial Organic Fertilizer Co. Ltd. (see Suppl. Table S1 for nutrient elements); (iv) natural loam soil + 10% (w/w) organic fertilizer + TOR3209 at the dose of 10^7^ CFU/g (TF). Strain TOR3209 was activated on agar plate of Gause’s No. 1 medium (g/L): soluble starch, 20.0; NaCl, 0.5; FeSO_4_·7H_2_O, 0.01; K_2_HPO_4_, 0.5; KNO_3_, 1.0; MgSO_4_·7H_2_O, 0.5; Agar 15.0; pH 7.0^1,8^. After incubation at 35 °C for 96 h, the activated TOR3209 on plate was washed off with 10 mL of sterile water and were transferred into 600 g bran solid medium^1,9^ (g/kg: wheat bran, 837; CaCO_3_, 7; NaCl, 2.5; KH_2_CO_3_, 2.5; KNO_3,_ 1.0; medical stone, 150; water, 600; autoclaved at 121 °C for 40 min). The inoculated medium was incubated at 35 °C for 120 h before being used as the inoculum. For each treatment, 22 pots (1 seedling per pot) were planted and all the pots were randomly arranged in equal watering. The tomato seedlings in pots were cultured in greenhouse under natural sunlight and temperature and distilled water was added when it is necessary.

The dry weight of tomato plants representing the growth traits was determined in four growth stages, including seedling stage (one month after the transplanting), flowering stage, early fruit setting stage, and late fruit setting stage. For this measure, three tomato plants were randomly destructive sampled for each treatment/each growth stage. Briefly, the plants were uprooted carefully from the pot and the roots, after rhizosphere soil sampling as mentioned subsequently, were washed with tap water to remove the attached soil. Then the plants were covered with a clean absorbent paper to eliminate the excessive water and were dried in an oven at 70 °C for 48 h. Finally, the dry plants were weighted to get their biomass in dry weight^20^. The tomato fruit yields (fresh weight) in different treatments were recorded only in the late fruit setting stage from 12 plants, including 3 used for plant dry weight measurement and 9 for yield measurement only. The total fruit mass of 4 randomly combined plants in each treatment was considered to represent the yield of the treatment, and the 12 plants were divided as three replicates. Significances of differences among the data of treatments were estimated by one-way ANOVA test, at a significance level of *P* < 0.05. The statistical analysis was performed using IBM SPSS Statistics 22.0 software program.

### Structural analysis of microbiome affected by TOR3209 inoculation

#### Sample collection and DNA extraction

For each sampling in the four growth stages described above, rhizosphere soils were sampled by brushing off the soils attached to the root surface with a soft toothbrush. Samples from the three plants (repeats) were compiled to form a composite sample and were stored at − 80 °C until it was used for DNA extraction^6^. The original soil without planting was included as bulk soil. The metagenomic DNA was extracted from 0.5 g of rhizosphere soil with three technical repeats (three subsample of the compiled sample) using the FastDNA SPIN Kit for Soil (MP Biomedicals, USA) according to the manufacture’s protocol. The yield and purity of extracted DNA were measured by Nanodrop 2000 spectrophotometer (Thermo Fisher Scientific, USA). And the integrity was detected by electrophoresis in 1% (w/v) agarose gel.

#### PCR amplification and Illumina high-throughput sequencing

In this analysis, the V3–V4 region of 16S rDNA was amplified from each technical repeat of the genomic DNA extracted from the rhizosphere soil samples, with specific primers with barcode: 341F (5′-CCT AYG GGR BGC ASC AG-3′) and 806R (5′-GGA CTA CNN GGG TAT CTA AT-3′) and the corresponding PCR protocol^21^. The PCR amplicons were visualized by electrophoresis in 1% (w/v) agarose gel and recovered (AMPure XP Beads, Beckman Coulter, USA), and quantified using Qubit3.0 fluorometer (Life Invitrogen, USA). The purified amplicons were mixed in equal amounts, connected to a sequencing adapter, and a sequencing library was constructed. Finally, the amplicons were sequenced using Illumina Hiseq 2500 Plateform (Illumina, USA) by the company of Gene Denovo.

#### Sequence analysis and statistics

To get high quality clean reads, raw reads were filtered according to the following rules: (i) removing reads containing more than 10% of unknown nucleotides (N); (ii) removing reads containing less than 80% of bases with quality (Q-value) > 20. The filtered reads were then assembled into tags according to overlap between paired-end reads with more than 10 bp overlap, and less than 2% mismatchs^22^. The software Mothur (v.1.34.0) was used to cluster tags of more than 97% identity into OTUs. The taxonomic classification of OTUs was based on annotation result of contained tags according to the mode principle, that is, the taxonomic rank which contained more than 66% of tags was thought to be the taxonomic rank of this OTU; otherwise the higher rank would be considered. The alpha diversity of OTUs, including Shannon index and Chao1 index and Shannon rarefaction curve, were estimated according to the instructions by Mothur^23^. Based on the OTU expression profile data, the pheatmap and gmodels package in R were used to make the heat map and PCA analysis, respectively^24,25^. The heatmap, Principal Component Analysis (PCA) and beta diversity analysis were performed to see the distribution of the OTUs with relative abundance ≥ 0.1 at least in one sample. The distance between samples was calculated and then cluster analysis was carried out using R package pvclust to predict the sample similarity on OTU level. Composition of the main microbial community among the treatments were analysed by Metastats and LEfSe^26,27^. Linear discriminant analysis (LDA) effect size (LEfSe) method was used to identify the most differentially abundant taxa between treatments which would help to discover the biomarkers. LEfSe with the Kruskal–Wallis rank sum test was applied to detect the features with significantly different abundances among the treatments. Next Wilcoxon rank sum test was used to detect the features with significantly different abundances between two treatments.

### Isolation and identification of cultivable bacteria affected by TOR3209 inoculation

#### In vitro screening of bacterial isolates affected by TOR3209

The tomato seeds of cv. Kaixuan and the substrate for culturing the tomato seedlings were the same as mentioned above. The tomato seeds was sterilized by washing three times with sterile water, soaking in 5% (v/v) sodium hypochlorite solution for 5 min, and soaking in 70% (v/v) ethanol for 5 min then washing 10 times with sterile water^2,8^. The *Streptomyces* sp*.* TOR3209 inoculum (prepared same as mentioned above) was evenly mixed with the seedling substrate at a concentration of 10^7^ CFU/g, and uniformly filled into a 32-well seedling tray (T, standards: 54 cm × 29 cm × 5 cm, 3 seedling trays per treatment group). The control was a blank substrate without adding bacteria (CK). The substrate was moistured by spraying an equal amount of water and cultured at a temperature of 25 ± 5 °C under nature sunlight in greenhouse.

After 60 days of growth, three healthy tomato seedlings with uniform sizes in the group T and group CK were gently removed from the substrate. Then 1.0 g of seedling roots were put in 20 mL 0.85% saline solution and were shaken at 200 rpm/min for 30 min to release the rhizosphere soil for obtaining a microbial suspension^2,7^. Then the roots were surface sterilized and grinded with 5 mL of physiological saline as the original extraction of root endophytes^28,29^. The original solutions of rhizosphere microbes and root endophytes were diluted to 10^–4^ and every dilution was plated onto NA4 plate (0.1 mL per plate), which contained (g/L): K_2_HPO_4,_ 1.0; KH_2_PO_4,_ 1.0; Sodium pyruvate, 0.5; peptone, 10.0; beef extract, 3.0; glucose, 3.0; NaCl, 5.0; agar, 15.0 and yeast, extract 1.0. After incubated at 30 °C for 48 h, colonies with different morphologies, especially those specific and dominant in the TOR3209 treatment were picked up for further purification. Subsequently, all the isolates were reserved in 20% (v/v) glycerol at − 80 °C and were tested for indole acetic acid (IAA) production, siderophore production and phosphate solubilization using the method of Benedetto et al*.*^30^, and the nitrogen fixation was tested using the method of Batista et al*.*^31^.

Genomic DNA was extracted from the isolates and used for amplifying the 16S rRNA gene with the primers 27F and 1492R and PCR protocol of Benedetto et al*.*^30^. The amplicons were checked by electrophoresis in 1% (w/v) agarose gel^32^ and were sequenced commercially with the same primers in Sangon Biotech Co. (Shanghai). The acquired sequences were used for blasting in NCBI data base to extract the most similar sequences. For identifying the isolates, the Software MAGA6 was used to construct phylogenetic tree with Neighbor-joining method with 1000 bootstrap replications for the acquired sequences and the similar sequences extracted from data base.

#### Tomato growth promotion of the bacterial isolates

##### Effects on germination and growth in sterile condition

Bacterial isolates were cultured separately in NA4 broth at 30 °C for 12 h with shaking (180 rpm) till the exponential phase, then the cultures were centrifuged and the bacterial cell pellets were washed three times with 0.85% saline solution. Finally, the pellets were suspended in the sterile water to obtain the concentration of 10^4^/10^5^ CFU/mL, and 4 mL of the prepared bacterial suspension were added to moisture the sterilized filter paper in sterile plate. For blank control, the same amount of sterile water was added. In each of the filter plates, 20 surface sterilized tomato seeds with similar sizes were evenly placed and incubated in the dark at 25 °C. After 7 days, the seedling length and number of germinated seeds were measured. Three biological replicates were included for all treatments. Germination rate (%) was calculated as 100 × number of germinated seeds/number of incubated seeds in the treatment. The growth promoting effect was judged as both the increase of germination rate and the seedling length.

##### Growth effect in greenhouse under semi natural condition

The bacterial suspension was mixed with 200 g of sterilized substrate (see “Pot experiment designation” and “In vitro screening of bacterial isolates affected by TOR3209” sections) at a final bacterial concentrations of 10^4^ CFU/g or 10^5^ CFU/g, which has been proved adequate for promoting growth based on the results of effects on germination and growth in sterile condition. The substrate (200 g) inoculated with bacteria was put in the 32-well nursery tray (standards: 54 cm × 29 cm × 5 cm) and 32 seeds were transplanted into each of the trays with distance of 5 cm between rows. After the seeds were covered with substrate, quantitative sterile water was poured to obtain 60% of the water retention capacity. The substrate without bacterial inoculation treated with the same amount of sterile water was used as a blank control. The two rows at the edge of the nursery tray were used as protection rows. The trays were cultured at 25 °C under natural sunlight (in April). The number of surviving seedlings and growth were regularly observed, and the emergence rates were recorded. Plants were sampled after 45 days of incubation for measuring their biomass and other indicators in the seedling stage to determine the effect of growth promotion in greenhouse.

#### PGP verification of bacteria affected by TOR3209 with pot experiment

The most representative bacterial strain affected by TOR3209 inoculation was selected for greenhouse pot experiment. Detailed preparations can be found in “Pot experiment designation” section. The experiments included 6 treatments: CK (natural loam soil, see “Pot experiment designation” section); T (CK + 10^7^ CFU/g TOR3209); F (CK + 10% organic fertilizer, see “Pot experiment designation” section); TF (T + 10% organic fertilizer); WF (F + 10^5^ CFU/g WSW007 bacterial suspension); WTF (TF + 10^5^ CFU/g WSW007 bacterial suspension). For each treatment, 20 pots were transplanted with 1 seedling per pot, in which 3 plants in each treatment were used for measuring biomass in the four growth stages as mentioned in “Isolation and identification of cultivable bacteria affected by TOR3209 inoculation” section. The fruit yield of 3 plants in the late fruit stage was also measured together with other 6 plants without biomass measurement. All seedlings were randomly arranged in greenhouse with natural conditions and equal watering. In each treatment, the yield of 3 plants was the total output of the treatment, and the plants were divided randomly into three replicates. The relative content of chlorophyll was determined by Chlorophyll meter SPAD-502 Plus (KONICA MINOLTA, JAPAN). To confirm the results of this verification tests, we repeated the same experiment in 2020 with 1 kg substrate for treatments CK, T, W, and WT. The plant dry weight was determined and rhizosphere soil was sampled every 15 days in triplicate (three plants). The rhizosphere soil were used for counting the number (CFU/g) of TOR3209 in tomato rhizosphere soil by serial dilution and plate spread method same as that used in “Isolation and identification of cultivable bacteria affected by TOR3209 inoculation” section on Gause’s No. 1 medium. The colonies of *Streptomyces* were recognized by their morphology and were identified by sequencing of the 16S rRNA gene after incubated at 35 °C for 4 days. Then we repeated the same experiment in 2020 for treatments F, TF, WF, and WTF. The fruit production was recorded in the late fruit stage, same as that mentioned above.

## Results

### Tomato biomass and yield in four growth periods

**Figure 1:**
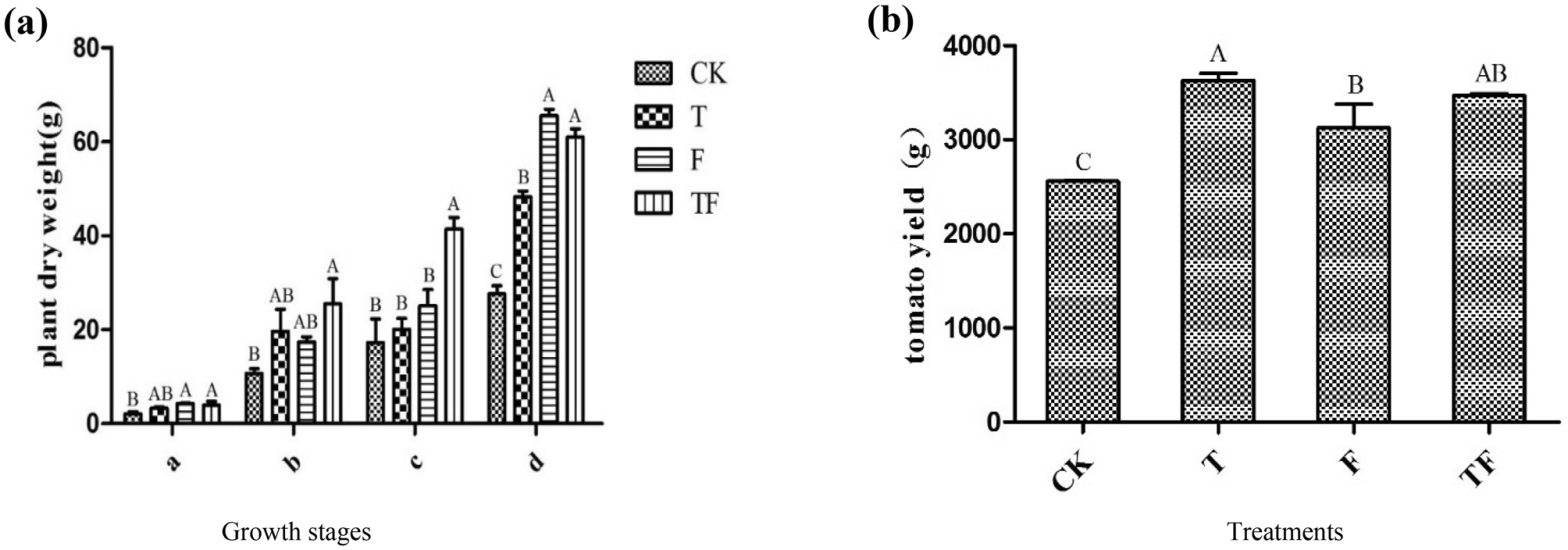
Effects of *Streptomyces* sp. TOR3209 inoculation on biomass and production of tomato. (**a**) plant dry weight of the four treatments at four growth periods: a, b, c, d represented seedling stage, flowering stage, early fruit setting stage, and late fruit setting stage, respectively. (**b**) Total yields (four plants) of the four treatments: CK, natural loam soil; T, soil + TOR3209 at dose of 10^7^ CFU/g; F, soil + 10% of organic fertilizer; TF, T + 10% of organic fertilizer. Significant differences (*P* < 0.05) among the four treatments were indicated by different capital letters on the top of columns.

Plant dry weights and total yields were shown in Fig. 1. As the life cycle advances, plant biomass increased continuously (Fig. 1a). In the four growth periods of the sampling, the dry weight of almost all the plants inoculated with TOR3209 (T and TF) were greater than that in corresponding controls without TOR3209 (CK and F), especially in the later growth stages. Except for the late fruit setting stage, there was no significant difference in the dry weight between the T and F treatments. As the most direct and important criterion for judging the growth promotion, the tomato yield (Fig. 1b) in treatment T (with TOR3209) was the highest, reaching 3632 g (4 plants), which was 1073 g (*P* < 0.05) greater than that (the lowest) in CK. The total yield was 3126 g in treatment F and was 3469 g in treatment TF. And the yield of the T treatment was 16.19% (*P* < 0.05) higher that of the F treatment.

### Diversity and community composition of rhizosphere microbiota

After quality filtering, a total of 3,170,705 sequences (raw tags) with an average length of 456 bp were obtained from the 51 rhizosphere soil samples. Based on the threshold of 97% sequence identity, these sequences were clustered into 6631–11,844 operational taxonomic units (OTUs) depending on the replicate and treatment, which presented coverages ≥ 90.85% (Table 1), and the OTU Shannon rarefaction curve (see Suppl. Fig. S1a) tended to be flat, suggesting that the sequencing depth of the samples can reflect the species diversity in the samples. The OTU numbers in the treatments without organic fertilizer addition (CK and T) were relatively high in the seedling and early fruit setting stages, which was significantly different from those in the flowering and later fruit setting stages. The numbers of OTU in the seedling stage was relatively high in the organic fertilizer treatments (F and TF), and there was no significant difference in the other three stages, which was consistent with the trend of the chao index (Table 1). In the four stages of tomato growth, the number of OTUs in treatment TF was relatively low compared to that in the other treatments. In the seedling stage, the chao values in the T and F treatments were higher than those in the other treatments, and there was no significant difference in the other three growth stages. Compared with the other treatments, the Shannon index was higher in CK in the four stages, indicating that the species in the CK were richer and had better uniformity. There was no significant difference in Shannon index between T and CK treatments during the flowering and early fruit setting. As shown in Suppl. Fig. S1, the three replicates in each treatment presented high reproducibility. Both the substrate treatments (with or without organic fertilizer) and the growth stages affected the distribution of OTUs. Heatmap results displayed that the CK was relatively separated from the other three treatments. In the flowering stage, treatments T and CK had the greater similarity. In the late fruit setting stage, the similarity of OTU expression profiles of the four treatments reached the highest (Suppl. Fig. S1b). All samples were separated according to the growth stage and organic fertilizer (Suppl. Fig. S1c). The organic fertilizer was apparently one of the influencing factors in the first principal-coordinate axis (PC1), and contributed 24.3% of the total variation, suggesting that the microbial community composition in rhizosphere of tomato was frequently related to the organic fertilizer. In beta diversity heatmap (Suppl. Fig. S1d), the smallest species diversity difference among the four treatments was observed in the seedling stage.

**Table 1:** The bacterial richness and diversity in tomato rhizosphere soil in different treatments at four growth stages.

Treatment*	Diversity parameters^#^			Coverage (%)
	OTU number	Chao	Shannon	
OS	7653^C^	13,497.02^C^	7.69^A^	92.05
aCK	10975^aA^	17,406.58^aBC^	7.97^aA^	94.72
bCK	8910^bcA^	16,548.22^aA^	7.72^abA^	92.38
cCK	9731^abA^	18,886.6^aA^	7.78^abA^	92.09
dCK	8528^bcA^	16,955.66^aA^	7.61^bA^	91.7
aT	10895^aA^	20,940.57^aAB^	7.66^aB^	93.78
bT	8086^bcAB^	14,313.52^aA^	7.61^aA^	92.96
cT	9855^abA^	22,902.03^aA^	7.75^aA^	90.85
dT	7062^cB^	12,415.59^aA^	7.46^aB^	92.74
aF	11844^aA^	23,370.35^aA^	7.82^aAB^	93.12
bF	7989^bAB^	14,612.8^bA^	7.39^bB^	93.6
cF	8153^bA^	16,784.45^bA^	7.48^bA^	92.27
dF	7523^bAB^	13486^bA^	7.56^bAB^	92.94
aTF	9435^aB^	17,431.7^aBC^	7.38^aC^	94.62
bTF	7539^bB^	14,232.18^bA^	7.33^aB^	93.47
cTF	7611^bA^	13,976.28^bA^	7.45^aA^	93.12
dTF	6631^bB^	12,219.68^bA^	7.296^aC^	93.30

*OS: original sample soil; CK: natural loam soil with tomato plant; T: soil + TOR3209 at the dose of 10^7^ CFU/g; F: soil + 10% organic fertilizer; TF: T + 10% organic fertilizer; letters a, b, c, and d in front of each treatment represent seedling stage, flowering stage, early fruit setting, and late fruit setting, respectively.

^#^Different superscript small letters following each number indicate significant differences among the growth periods in the same treatment; the capital letters indicate significant differences among the treatments in the same growth stage. The comparison of OS and different treatment groups was at seedling stage.

### Bacterial community composition and abundance in rhizosphere

**Figure 2:**
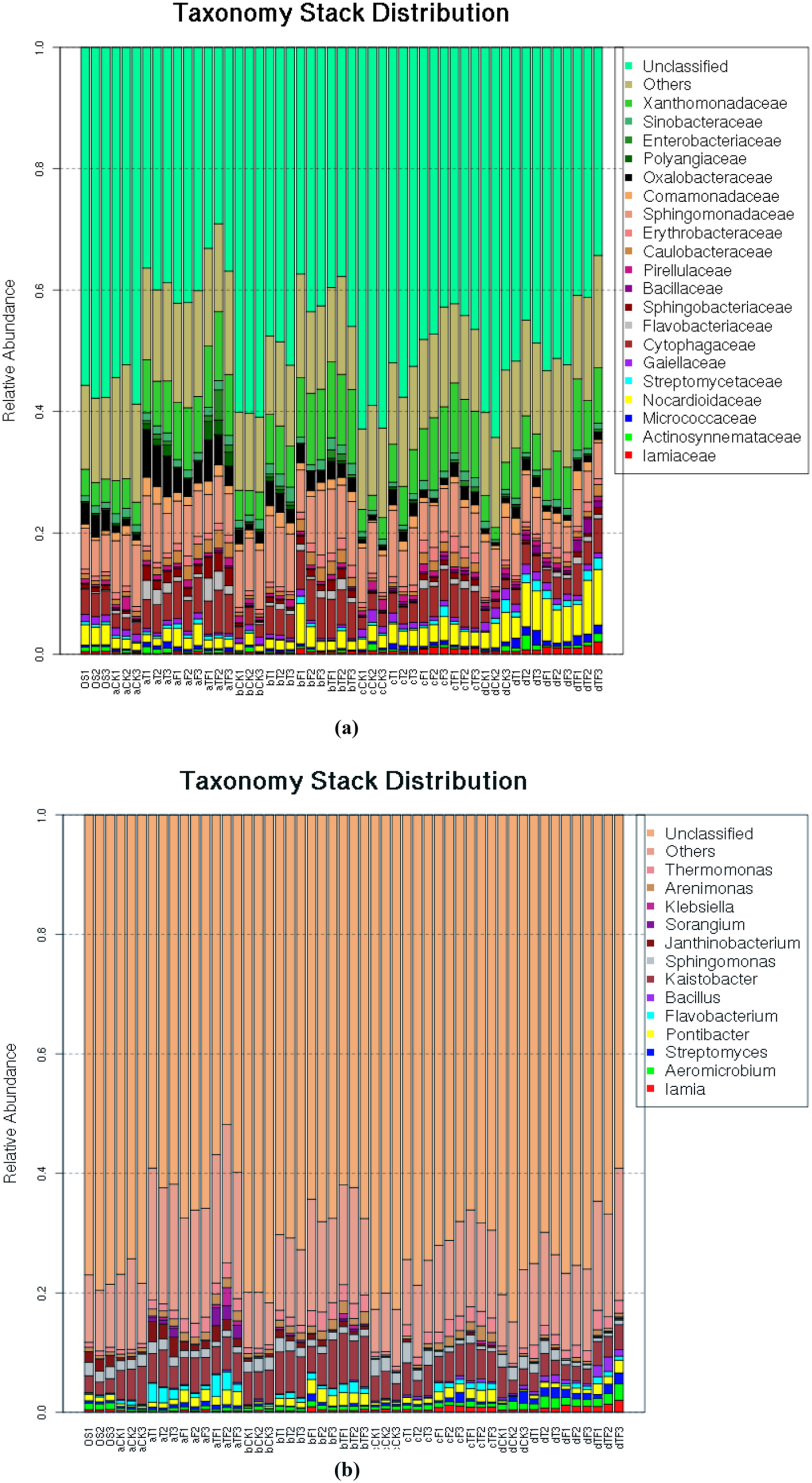
Relative abundances of bacterial taxa identified at the family (**a**) and genus (**b**) levels for each tomato rhizosphere soil sample.

Combining the taxonomic annotation and distribution of OTUs in different samples, the distribution of bacterial family and genus in the treatments were estimated for the OTUs with relative abundance ≥ 2% in at least one sample. The OTUs corresponding to the defined taxa with relative abundances < 2% were classified as the Other category; while the OTUs that cannot be annotated to defined family and genus were defined as the Unclassified category. As shown in Fig. 2, the soil samples with similar bacterial compositions showed varied abundances for the same family and genus. A total of twenty bacterial families with > 2% of abundance were identified among different samples, in which *Xanthomonadaceae*, *Sphingomonadaceae, Cytophagaceae, Nocardioidaceae,* and *Oxalobacteraceae* were predominant. The abundances of *Iamiaceae* and *Nocardioidaceae* in all treatments were gradually increased with the development of tomato plants (Fig. 2a). The proportion of *Bacillaceae* was small in all treatments. And the relative abundances of families *Flavobacteriaceae*, *Sphingobacteriaceae, Polyangiaceae* and *Enterobacteriaceae* in treatments T and TF were greater than that in treatments CK and F, respectively; while the situation of *Gaiellaceae* was contrary. At the genus level (Fig. 2b), *Janthinobacterium* made up the majority, and in treatments with TOR3209 inoculation (T and TF) the abundances of *Flavobacterium*, *Janthinobacterium*, *Sorangium* and *Klebsiella* were increased. In addition, the abundances of *Sorangium* and *Klebsiella* in the treatments without TOR3209 (CK and F) were very small (even zero). Changes in bacterial abundance and structure suggested that TOR3209 inoculation as well as growth stages modified the microbial community in tomato rhizosphere and affected the growth and development of tomato.

### Effect of TOR3209 inoculation on function and abundance of representative bacteria

In linear discriminant analysis (LDA) on effect size (LEfSe), the treatments CK and F were used as the controls for treatments T and TF, respectively. The LEfSe method was used for quantitatively comparative analysis of rhizosphere bacterial taxa between CK and T (Suppl. Fig. S2) and between F and TF (Suppl. Fig. S3) for further elucidating the possible interactions of the biomarkers with TOR3209. In general, the taxa as biomarkers were selected with *P* < 0.05 and LDA score (the impact size of different taxa), depending on the plant growth stage (Suppl. Tables S5–S12). The numbers of taxa with significantly different abundance in CK and T were gradually decrease with the development of tomato plants, while they were greater in seedling stage and late fruit setting stage compared to that in the other two stages in F and TF (Suppl. Figs. S2, S3, Tables S5–S12). The cladogram revealed 252 biomarkers in CK and T in seedling stage. Across all the taxonomic levels in CK and T, 134 biomarkers were associated with CK and 118 with T treatment (Suppl. Fig. S2a). The greater abundances of *Proteobacteria* (LDA > 4.48, *P* < 0.05) and *Bacteroidetes*, including families *Oxalobacteraceae*, *Xanthomonadaceae*, *Cytophagaceae* and *Flavobacteriaceae,* were significantly enriched in treatment T in seedling stage. The microbes in *Acidobacteria* and *Actinobacteria* were evidently abundant in CK while *Proteobacteria* and *Bacteroidetes* were in bT group (T in the flowering stage) (Suppl. Fig. S2b, LDA > 4, *P* < 0.05). When the LDA > 3.5 in early fruit setting stage (c), the families *Xanthomonadaceae* and *Sphingomonadaceae* were dominant in group cT. Besides the family *Xanthomonadaceae*, families *Cytophagaceae*, *Micrococcaceae* and *Oxalobacteraceae* were richer in treatment dT (late fruit setting stage) than that in treatment dCK (LDA > 3.5, *P* < 0.05).

As shown in Suppl. Fig. S3a, out of the nineteen different bacterial taxa, sixteen, including *Sorangium cellulosum,* were abundant in the TF treatment (LDA score > 4.0, *P* < 0.05). When LDA score is greater than 3.5 in the flowering stage, only seven taxa presented significant difference, in which the order *Acidimicrobiales* was recorded in F treatment, while *Bacteroidales* and *Enterobacteriales* were found in TF (Suppl. Fig. S3b). The differentially abundant taxa in the rhizosphere soils of early fruit setting stage were families *Oxalobacteraceae* and *Enterobacteriaceae* (LDA score > 3.5, *P* < 0.05, Suppl. Fig. S3c). The dramatically enriched genera in TF were *Bacillus*, *Kaistobacter* and *Pontibacter*. On the contrary, five class were predominant in TF treatment, including *Acidobacteria*, *Gemmatimonadetes*, *Phycisphaerae*, *Betaproteobacteria* and *Deltaproteobacteria* (LDA score > 3.5, *P* < 0.05, Suppl. Fig. S3d). All the results indicate that dominant microbes in different developmental stages and treatments are distinct, which may be favorable for special promoting growth mechanisms in tomato.

**Figure 3:**
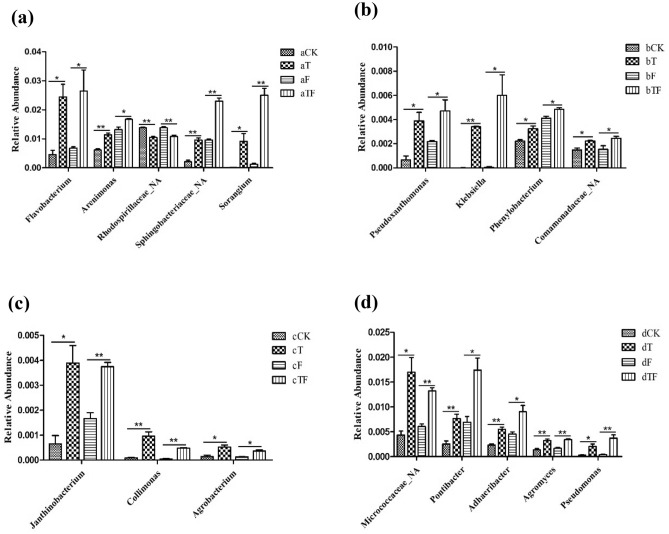
Genera with relatively large abundance that were affected by TOR3209 inoculation. (**a**) seedling stage; (**b**) flowering stage; (**c**) early fruit setting stage; (**d**) late fruit setting stage respectively.

In order to further find the possible related communities affected by TOR3209 inoculation, the common genera with significant difference (*P* < 0.05) between CK and T and between F and TF treatments were screened according to the data of Metastats. At the genus level, the number of bacteria affected by TOR3209 in the four growth stages was 37, 9, 10, and 9, respectively. In each stage, the genera with higher relative abundance were selected for analysis. As shown in Fig. 3, the marked bacteria with significant differences in each stage were different, and most of them were dominant in the treatments containing TOR3209. In seedling stage, except *Rhodospirillaceae*_NA, the other four bacteria (*Flavobacterium*, *Arenimona*, *Sphingobacteriaceae*_NA and *Sorangium*) were more dominant in T and TF than those in the control treatments CK and F, respectively (Fig. 3a). In flowering stage, the genus *Klebsiella* was found only in treatments with TOR3209 (T and TF); meanwhile, *Pseudoxanthomonas, Phenylobacterium* and *Comamonadaceae_*NA were more dominant in T and TF than in the controls (CK and F) (Fig. 3b). In the early fruit setting stage, the abundance of *Janthinobacterium* was significantly increased by inoculation of TOR3209; while *Collimonas* and *Agrobacterium* were with a very low abundance in the control group (CK and F) (Fig. 3c). Five genera (*Micrococcacaea*_NA, *Pontibacter, Adhaeribacter, Agromyces* and *Pseudomonas*) were the representatives in the late fruit setting stage, in which *Pseudomonas* was very little in treatments CK and F, and the other four were more dominant in T and TF than in the controls (CK and F) (Fig. 3d). These results clearly proved that the community structure and abundances of some bacterial taxa in rhizosphere were affected by inoculation of TOR3209 and by growth stages of tomato.

### Identification and characterization of bacteria affected by TOR3209 inoculation

Compared to that in the controls, the fresh weight and dry weight of the tomato seedlings in the TOR3209 inoculation treatments increased by 51.5% and 112.0%, respectively. The isolation results of rhizosphere soil and root endophytes from CK and T samples showed that the similarity of cultivable bacteria was high between these two treatments. Compared with CK, there were only four unique or dominant strains (WSW001, WSW002, WSW003 and WSW004) in rhizosphere of T treatment and they were identified by phylogeny of 16S rDNA as *Enterobacter* sp*.*, *Arthrobacter* sp., *Bacillus subtilis* and *Rhizobium* sp*.*, respectively (Suppl. Table S2, Fig. S4). For endophytes in root, only a bacterium named *B. velezensis* WSW007 was unique in T treatment, which reached to 1.5 × 10^5^ CFU/g while it is almost absent in CK. The PGP characteristics of these five isolates were different. All of them could fix nitrogen, while *Enterobacter* sp*.* WSW001 and *Rhizobium* sp*.* WSW004 produced siderophore. None of them dissolved inorganic phosphorus, but WSW001 dissolved organic phosphorus. All strains produced IAA, with *Rhizobium* sp*.* WSW004 the highest producer (2.534 mg/L, Suppl. Table S2).

### Growth promoting effects of bacteria affected by TOR3209 inoculation

**Figure 4:**
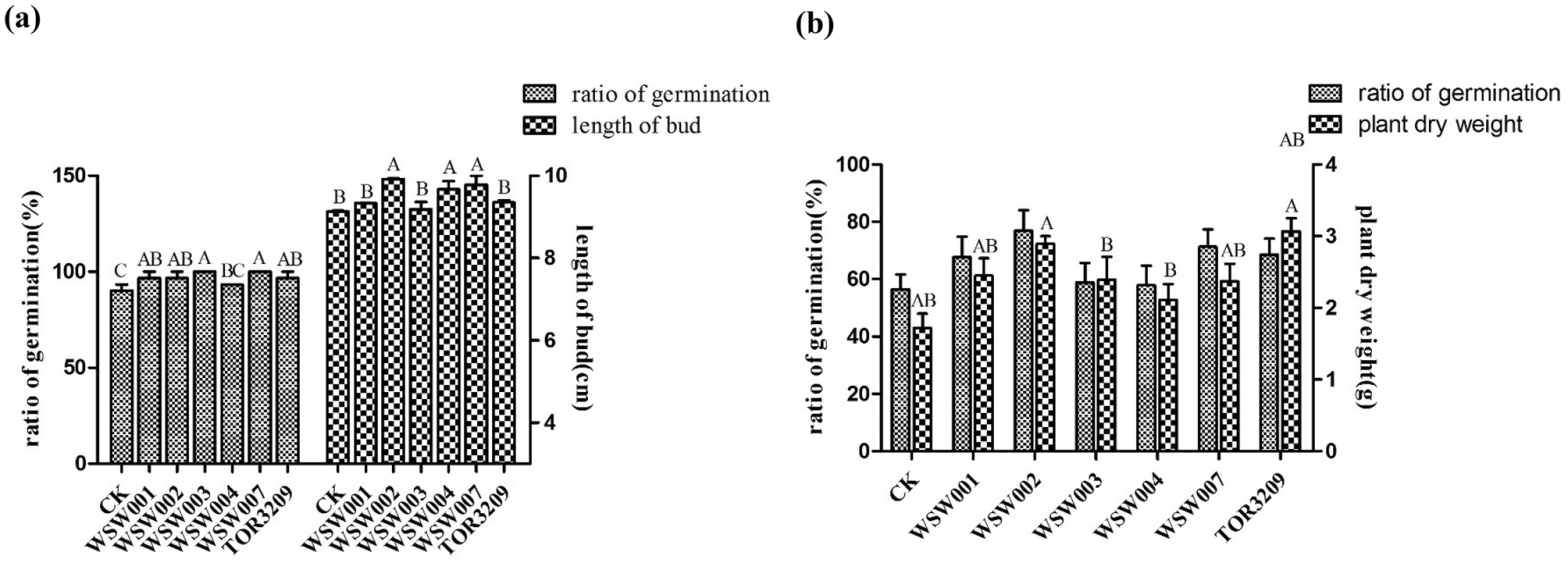
Results of growth promotion experiment of isolates affected by TOR3209 inoculation. (**a**) Indoor aseptic germination test; (**b**) tomato biomass after 7 days of pot experiment in greenhouse. Different letters representing significant differences (*P* < 0.05).

The strains WSW001, WSW002, WSW007 (at 10^5^ CFU/mL) and WSW003, WSW004 (at 10^4^ CFU/mL) presented better effects on growth promotion in the indoor sterile conditions (Fig. 4a, Suppl. Table S13). The germination rate of seeds treated with WSW003 and WSW007 was 100%, while that of the control was 90%. The length of germinated seedling treated with WSW007 was 9.78 ± 0.22 cm, which was 0.65 ± 0.05 cm higher than that of the control group.

According to the results of aseptic germination experiment, the corresponding concentration of bacterial suspension was selected for greenhouse pot experiment (Fig. 4b, Suppl. Table S13). After 7 days of sowing, the germination rate of tomato seeds treated with *Arthrobacter* sp*.* WSW002 was the highest (76.85%), which was 20.37% greater than that of the control group; the second highest germination rate was observed in the seeds treated with strain WSW007. However, there was no significant difference in germination rate among all the treatments (*P* > 0.05). After 30 days of sowing, the dry weight of tomato seedlings in the treatment groups was higher than that in the control group, in which the *Arthrobacter* sp*.* WSW002 treated group was the highest with 2.89 ± 0.11 g. In summary, all the five strains presented a stimulating effect on tomato germination and seedling growth, in which *Arthrobacter* sp*.* WSW002 had the strongest comprehensive ability, followed by *B. velezensis* WSW007. These strains may work synergistically with TOR3209 to promote tomato growth.

### Synergistically PGP effects between strains WSW007 and TOR3209

**Figure 5:**
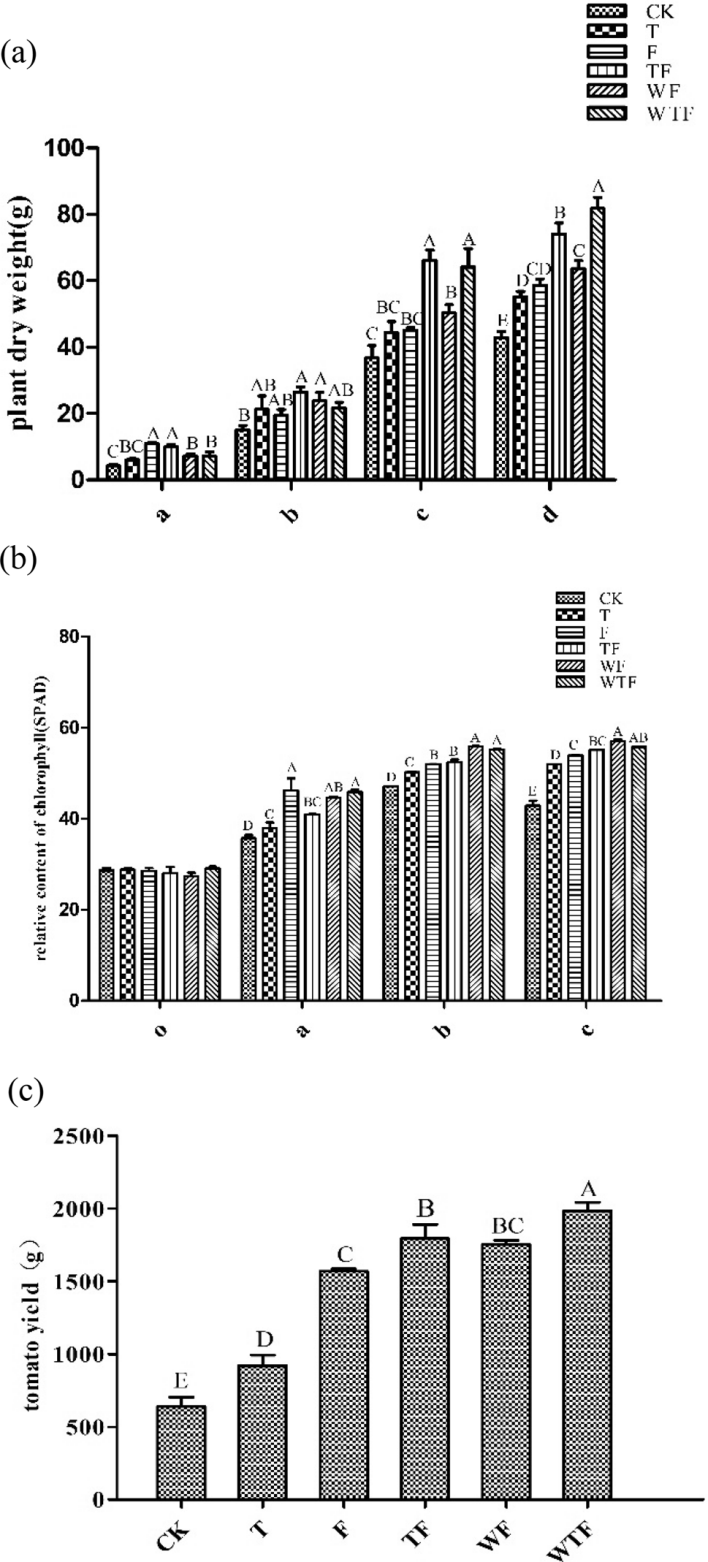
Effects of inoculation with *B. velezensis* WSW007 and/or *Streptomyces* sp. TOR3209 on tomato plants in outdoor pot experiments. (**a**) Plant dry weight at four different growth stages; (**b**) relative content of chlorophyll at four different growth stages; (**c**) total yield (three plants) from six different treatment groups. Letters a, b, c, and d represent seedling stage, flowering stage, early fruit setting stage, and late fruit setting stage, respectively; O represents the initial period. CK: natural soil; T: soil + TOR3209; F: soil + 10% (w/w) organic fertilizer; TF: soil + 10% organic fertilizer + TOR3209; WF: F treatment + WSW007; WTF: TF + WSW007. Different letters representing significant differences (*P* < 0.05).

*Bacillus velezensis* WSW007 was the only endophyte in root with significantly increased abundance responding to TOR3209 inoculation. Based on this, WSW007 and TOR3209 were used to test their cooperation in pot experiment. The results showed that the bacterial inoculation treatments presented higher tomato plant biomass than that without bacterial inoculation, and the biomass was significantly higher in treatments of inoculation with *Streptomyces* sp. TOR3209 + organic fertilizer (TF) and with combined *B. velezensis* WSW007 and TOR3209 + organic fertilizer (WTF) than that in CK and in the other treatments in the early and late fruit setting stages (Fig. 5a), demonstrating that TOR3209 has the ability to enhance the biomass. While the biomass in WTF was also significantly greater than that in TF in the late fruit setting stage, evidencing the synergistical effects between the two inoculant strains. The chlorophyll content of plants in the treatment inoculated with WSW007 (WF and WTF) were higher, suggesting that its inoculation might promote the photosynthesis efficiency of the plants (Fig. 5b). As shown in Fig. 5c, treatment WTF had the highest tomato yield among all the treatments that evidenced again the positive cooperation between WSW007 and TOR3209. In the confirmation experiment in 2020, the results once again proved the growth promotion and production increase by TOR3209 and WSW007 alone, and by combination of these two strains (Suppl. Figs. S5, S6). All these results demonstrated that the growth promoting effect was better in treatment of co-inoculation of WSW007 and TOR3209.

### Dynamics of TOR3209 inoculant in tomato rhizosphere

In this analysis, the number of viable cells of TOR3209 gradually decreased as time goes by (Suppl. Fig. S7). We added 10^7^ CFU/g TOR3209 while transplanting the tomato seedlings, and the number of viable cells was only 2.3 × 10^2^ CFU/g in the rhizosphere after 60 days of plant growth.

## Discussion

Growth promoting rhizobacteria are widely concerned because of their sustainable characteristics in agriculture. *Streptomyces* is a kind of well known PGPR based on its antimicrobial abilities against phytopathogens and plant growth-promoting features for wheat^33^, rice^34^, and tomato^35,36^, demonstrating it a PGPR with wide plant species. In our study, *Streptomyces* sp. TOR3209 could increase the dry weight of tomato seedlings at different growth stages (Figs. 1, 4, 5). The 78.5% increase of the dry weight of tomato seedlings by TOR3209 (Fig. 4) was greater than that of *Streptomyces* strain PM3 (26.8%)^35^, but lower than that of *Streptomyces fradiae* NKZ-259 (83.3%)^36^. However, the significantly increase of tomato yield by TOR3209 inoculation (nearly 41% in T and 10.97% in TF) compared with the corresponding controls (CK and F) demonstrated that strain *Streptomyces* sp. TOR3209 might improved both the nutrient supply (in T) and carbohydrate accumulation (in T and TF). In addition, this strain could also enhance the resistance of plants to pathogens^15,16^. Compared with the previously reported tomato PGPR, such as *Burkholderia* sp. L2^3,7^ and *Bacillus* spp. BETS11 and BETR11^3,8^, *Streptomyces* sp. TOR3209 has greater application potential in increasing agricultural yield based on its multiple impacts on tomato and its great reproduction rate by conidia generation in solid medium.

Up to date, few reports have been published about the interaction between *Streptomyces* and other rhizosphere microorganisms in the plant growth promotion. So it is interesting to find how *Streptomyces* strains change the rhizosphere microbial community. In the present study, the bacterial richness and diversity in tomato rhizosphere in four growth stages were not different, and little difference between the treatments with/without TOR3209 inoculation (Table 1). The changes in the microbial community structures caused by inoculation of TOR3209 (Fig. 2, Suppl. Figs. S2, S3) were similar to the results in a study on peanut rhizosphere^26^, implying that TOR3209 could modify the microbial community in rhizosphere and in turn to achieve the growth promotion effects, similar to the effects of strain *Pseudomonas fluorescens* pc78 on microbial community in tomato rhizosphere^39^.

Organic fertilizer is another major factor to change the microbial community structure in our study (Suppl. Fig. S1b) and in a previous study^40^. Organic fertilizer plays an important role in the growth of plants^41^. In our research, organic fertilizer has greatly changed the microbial community structure (Suppl. Fig. S1b). The results undoubtedly show that organic fertilizer is one of the key factors affecting the microbial community (Suppl. Fig. S1c). The addition of organic fertilizer will change soil physical and chemical properties, and also improve soil enzyme activity^42^. These changes in soil environment are closely related to the structure of microorganisms^43,44^. Besides, the application of organic fertilizer will inevitably affect the nutrient content of soil. Therefore, it has a significant impact on the abundance and community composition of the microorganisms using different nutrients^45^. Compared with organic fertilizer and growth period, TOR3209 has less effect on the whole microbial community, but its inoculation enhanced abundances of some beneficial bacteria and decreased abundances of the pathogenic bacteria in rhizobiome.

The existence of similar bacterial taxa in the rhizosphere soil samples from different treatments and growth stages in our study might reflects the strong selection of tomato plants on their rhizobiome, but the variation in relative abundances of the bacterial taxa at family and genus level (Fig. 2) might be related to the effects of TOR3209 inoculation and plant growth in the rhizobacteria. The enhance of relative abundances of families *Flavobacteriaceae*, *Sphingobacteriaceae*, *Polyangiaceae* and *Enterobacteriaceae,* and the decrease of *Gaiellaceae* in treatments T and TF compared with CK and F, respectively, fully evidenced the regulation effects of TOR3209 inoculation on rhizobiome (Fig. 2a). At the genus level, the increased abundances of *Flavobacterium, Janthinobacterium*, *Sorangium* and *Klebsiella* in treatments with inoculation of TOR3209 (T and TF), and the less existence and absence of *Sorangium* and *Klebsiella* in the treatments without TOR3209 (CK and F) (Fig. 2b) made us some possible explain for the mechanisms how TOR3209 improved the tomato growth and production. Among the up regulated genera by inoculation of TOR3209*, Sorangium* contained members with anti-tumor and anti-hypertension functions^46,47^, presenting strong ability to degrade fibers and abundant secondary metabolites, including antifungal substances^4,8^. The genus *Klebsiella* harbored strains with various PGP traits, including phosphorus solubilization and nitrogen fixation^49–51^. *Flavobacterium* is famous with their ability to degrade complex organic compounds and presents various PGP traints, like enhancing resistance to phytopathogens and producing phytohormones^52,53^. *Janthinobacterium* is a genus presenting antibiotic activity to the G^+^ bacteria and nitrogen fixing ability^5,4^, and it has been isolated from rhizopsphere of some crops^5,5^. Based on the functions of these bacteria, it could be estimated that the inoculation of TOR3209 stimulated the PGPR of tomato, which in turn enhanced the growth of plant by improving nutrient supplement (P and N), producing phytohormones, and inhibiting the phytopathogens.

The analyses of LEfSe and Metastats further verified the functional and representative bacteria responding to the TOR3209 inoculation. The research by Jaiswal et al*.*^56^ found biochar significantly stimulated the abundance of genera affiliated with soil-borne disease suppression, plant growth promotion, and/or biological N_2_ fixation, most notably: *Rhodanobacter*, *Sphingobium*, *Rhizobium*, *Pseudomonas*, *Achromobacter*, *Stenotrophomonas*, *Shinella*, *Bacillus* and *Flavobacterium* and so on. Some of these genera were detected as up regulated bacteria by inoculation of TOR3209 in our present study. In seedling stage, the greater dominance of *Rhodospirillaceae*_NA, *Flavobacterium*, *Arenimonas* and *Sphingobacteriaceae*_NA in the TOR3209 inoculation treatments (T and TF, Fig. 3a) implied that they may play roles in PGP processes. The members of *Rhodospirillaceae* are anaerobic photosynthetic bacteria, which can degrade the organic substances^57^. *Flavobacterium* may have the antibacterial activity as reported previously^52,53,58^. So far the genus *Arenimonas* contained seven species isolated from seashore sand, oil-contaminated soil, rice rhizosphere, compost, iron mine, compost and sediment of a eutrophic reservoir^59^. However, their effects on plants need to be explored. *Sphingobacteriaceae* is widespread in soils^60,61^ and may have strong adaptability in varied environments. The great enrichment of genus *Klebsiella* in flowering stage in TOR3209 inoculation treatments (Fig. 3b) might improving the nitrogen nutrient supply of the plant, since *Klebsiella pneumoniae* is a well known rhizosphere N_2_-fixer^49–51^. As the bacteria enriched by TOR3209 inoculation in the early fruit setting stage, *Janthinobacterium* and *Collimonas* are well known for their antifungal effects^62–64^, while *Agrobacterium* might be phytopathogen, endophytes or symbionts of plants depending the strain-specific properties^65,66^. In the last growth stage, TOR3209 inoculation increased the abundance of *Micrococcaceae*_NA, *Agromyces*, *Adhaeribacter*, *Pontibacter* and *Pseudomonas*, which formed microbial composition similar to that reported by Gao et al.^67^, suggesting them a biochar-shifted soil bacterial community favorably contributing to the resistance of tomato plants against bacterial wilt. In general, the microbes in rhizobacterial community up-regulated by TOR3209 were mainly the plant growth promoting and disease-resistant bacteria. Although the abundance of each genus varied in tomato rhizosphere soil samples with TOR3209 inoculation in different developing stages, all the results revealed that dominant microbes in different developing stages were distinct, which may be favorable for plant promotion mechanisms in tomato.

Curiously, the no dominant existence of *Streptomyces* genus in these growth stages implies that TOR3209 does not function through cell density but may serve as a regulator of microbial composition. Both our results and that of Kang et al.^62^ break the traditional view that establishment and maintenance of critical population densities in the rhizosphere was the premise of PGPR to exert growth-promoting effects. Therefore, we explored the variation of TOR3209 in tomato rhizosphere soil every 15 days. The result was what we expected (Suppl. Fig. S7) since TOR3209 was inoculated at the dose of 10^7^ CFU/g while transplanting tomato seedlings, and only 2.3 × 10^2^ CFU/g were detected after 60 days. This result implies that TOR3209 played its corresponding role in the initial stage which was not consistent with other PGP bacteria colonized in plant^68,69^. However, in addition to changing the microbial community structure, we need to further explore the growth promoting effect by TOR3209 strain on tomato (Fig. 4). There are many PGPR that can stimulate or regulate growth genes of plant^70^ or produce growth promoters such as IAA by strain itself or promote nutrient absorption of plants to achieve the purpose of promoting plant growth^71^. We need to further confirm whether TOR3209 is also through the above mechanisms.

In order to more fully explain the regulatory effect of TOR3209 on rhizosphere bacteria, four rhizobacteria identified as *Enterobacter* sp*.*, *Arthrobacter* sp., *Bacillus subtilis* and *Rhizobium* sp*.* and a root endophyte *B. velezensis* were isolated as bacteria up-regulated by TOR3209. These five strains have great potential in promoting plant growth (Fig. 4) and a large number of studies have proved the growth promoting ability of related strains^67,72–76^. The dominant differential strains screened by non-culture methods (Fig. 3a) are different from the cultured strains, because most of the dominants detected by the molecular methods are difficult for isolation, such as *Sorangium cellulosum*^77^.

It is worth noting that *B. velezensis* WSW007 was isolated as the dominant strain only in the tomato root treated with TOR3209 while it was rare in the CK. Generally speaking, PGPR's promoting effect on plants is the result of multiple mechanisms and rarely by a single way^78^. Besides, the way of these effects will change according to the environment. Indoor experiments are more artificial way to show the plant growth promoting properties, so we performed the outdoor pot experiments to reveal the effects of WSW007 on tomatoes. Pleasingly, the inoculation of WSW007 increased tomato yield by 11.72% (*P* < 0.05) compared to the control. As shown in Fig. 5, the yield of tomato in treatments of WF and TF was lower than that of WTF. It is clear that the strains WSW007 and TOR3209 promoted plant growth together (Fig. 5, Suppl. Figs. S5, S6). Our previous research found that WSW007 can enter the root from outside soil, so we speculate that WSW007 plays a role within the root. The effect of single strain on plant is limited, and the ideal PGP effect can be achieved by using the mutually beneficial symbiosis or functional complementarity between bacteria^79^. Therefore, screening and combination of composite strains with excellent properties is one of the research directions of microbial fertilizer at present. *B. velezensis* WSW007 and *Streptomyces* sp*.* TOR3209 could be candidates as microbial fertilizer. And mechanism of interaction between these two strains need to be explained. Moreover, our isolated probiotics can be combined to make microbial organic fertilizers.

Many studies have reported that *Streptomyces* can produce abundant metabolites, which play various roles in agriculture such as plant–promoting and against phytopathogens^80,81^. The wheat bran was used as a low-cost medium in the present study under solid-state fermentation (SSF) and the metabolites may affect the growth of tomato. Under the condition of solid-state fermentation based on bran, *Streptomyces* can produce metabolites including cellulase and natamycin^82,83^. These metabolites may play some kind role in plant growth and may change the microbial community structure. Therefore, it is one of the keys to study the growth promoting mechanism of TOR3209 by clarifying the *Streptomyces* strain itself or its metabolites. At the same time, it also provides another way to study the interaction between bacteria.

Conclusively, *Streptomyces* sp*.* TOR3209 could significantly promote the growth and enhance the yield of tomato by changing the microbial community structure in tomato rhizosphere. In addition, TOR3209 could recruit a strain of *B. velezensis* WSW007 into the root, and both of them promoted the growth of tomato. The strain WSW007 was a key response factor in the process of action by TOR3209. This research further revealed the complexity of microbe-plant interaction and provided a certain theoretical basis for the application of TOR3209 and WSW007 in the production of microbial organic fertilizers. However, further studies are needed to (1) dig deeper into functional potential of rhizosphere microbial community affected by TOR3209; and (2) explain how WSW007 responds to inoculation of TOR3209 and how these two bacteria interact with plants.

## Supplementary information

Supplementary Information.

## Data Availability

These sequence data have been submitted to the SRA databases under accession number PRJNA610155.
