# Supplementary material for: Streptomyces sp. strain TOR3209: a rhizosphere bacterium promoting growth of tomato by affecting the rhizosphere microbial community
Source: Sci Rep. 2020 Nov 18;10:20132. doi: 10.1038/s41598-020-76887-5 (PMC7675979; doi:10.1038/s41598-020-76887-5)
Supplement: Supplementary file 1 — Supplementary Information. [file 41598_2020_76887_MOESM1_ESM.docx]

*Streptomyces* sp. Strain TOR3209: a Rhizosphere Bacterium Promoting Growth of Tomato by Affecting the Rhizosphere Microbial Community

Dong Hu^1#^, Shuhong Li^1#^, Ying Li^1^, Jieli Peng^1^, Xiaoyan Wei^1^, Jia Ma^1^, Cuimian Zhang^1^, Nan Jia^1^, Entao Wang^2^, Zhanwu Wang^1*^

^1^Key laboratory of plants genetic engineering center, Institute of Genetics and Physiology (Hebei agricultural products quality and safety research center), Hebei academy of agriculture and forestry sciences, Shijiazhuang, Hebei, 050000, P. R. China.

^2^Departamento de Microbiología, Escuela Nacional de Ciencias Biológicas, Instituto Politécnico Nacional, C.P. 11340, Ciudad de México, México

#These authors contributed equally to this work.

***Correspondence author**

Email: zhanwuw@126.com

**SUPPORTING INFORMATION** online

**Table S1**. The major nutrient contents in different substrates for plant growth used in the study

| Samples | Total nitrogen (g/kg) | Total phosphorus (g/kg) | Total  potassium (g/kg) | Organic  matter (g/kg) | pH |
| --- | --- | --- | --- | --- | --- |
| Seedling substrate | 18.72 | 8.33 | 18.87 | 230.76 | 5.08 |
| Natural loam soil | 1.20 | 0.97 | 20.48 | 22.99 | 7.74 |
| Organic fertilizer | 14.70 | 31.30 | 20.00 | 343.70 | 7.49 |

**Table S2**. Identification and PGP characteristics of the selected rhizosphere or endosphere isolates up-regulated by inoculation of *Streptomyces* sp. strain TOR3209

| Strain number | Species affiliation by 16S rDNA | Origin | Nitrogen fixation | Organic phosphate solubilizing | Inorganic phosphate solubilizing | Production of IAA (mg/L) | Siderophore production |
| --- | --- | --- | --- | --- | --- | --- | --- |
| WSW001 | *Enterobacter* sp*.* | Rhizosphere | + | + | - | 0.153 | + |
| WSW002 | *Arthrobacter* sp*.* | Rhizosphere | + | - | - | 1.236 | - |
| WSW003 | *Bacillus subtilis* | Rhizosphere | + | - | - | 1.326 | - |
| WSW004 | *Rhizobium* sp*.* | Rhizosphere | + | - | - | 2.534 | + |
| WSW007 | *Bacillus amyloliquefaciens* | Endospere | *+* | - | - | 0.534 | - |

**Table S3.** Abundance of bacterial families in different treatments (CK, T, F, TF) and different growth stages (a, b, c, d) of tomato drawn in Figure 2a (in triplicate)

| **Bacterial Family** | **Copy number of 16S rDNA in seedling stage (a) of treatment** | | | | | | | | | | | |  |
| --- | --- | --- | --- | --- | --- | --- | --- | --- | --- | --- | --- | --- | --- |
|  | **aCK1** | **aCK2** | **aCK3** | **aT1** | **aT2** | **aT3** | **aF1** | **aF2** | **aF3** | **aTF1** | **aTF2** | **aTF3** |  |
| *Iamiaceae* | 294 | 202 | 229 | 129 | 100 | 206 | 395 | 226 | 433 | 217 | 193 | 270 |  |
| *Actinosynnemataceae* | 183 | 393 | 142 | 1032 | 613 | 671 | 165 | 212 | 324 | 307 | 356 | 154 | |
| *Micrococcaceae* | 310 | 212 | 204 | 660 | 506 | 927 | 510 | 310 | 508 | 321 | 212 | 544 | |
| *Nocardioidaceae* | 1478 | 1469 | 1049 | 1148 | 1026 | 1880 | 2578 | 1710 | 3089 | 1236 | 1423 | 1209 | |
| *Streptomycetaceae* | 345 | 404 | 180 | 429 | 350 | 487 | 665 | 483 | 700 | 558 | 392 | 420 | |
| *Gaiellaceae* | 1076 | 968 | 1090 | 530 | 455 | 646 | 730 | 566 | 860 | 287 | 270 | 392 | |
| *Cytophagaceae* | 1935 | 1777 | 1675 | 4284 | 3983 | 3864 | 5244 | 4590 | 5030 | 4734 | 5647 | 5685 | |
| *Flavobacteriaceae* | 444 | 612 | 152 | 2889 | 2150 | 1457 | 651 | 657 | 531 | 3289 | 2495 | 1234 | |
| *Sphingobacteriaceae* | 236 | 394 | 170 | 2194 | 1229 | 1633 | 1175 | 1203 | 1211 | 2988 | 2474 | 2593 | |
| *Bacillaceae* | 184 | 154 | 165 | 105 | 124 | 149 | 189 | 124 | 180 | 145 | 291 | 268 | |
| *Pirellulaceae* | 408 | 2014 | 382 | 669 | 718 | 665 | 536 | 1103 | 757 | 512 | 284 | 1198 | |
| *Caulobacteraceae* | 742 | 721 | 738 | 1443 | 1005 | 1106 | 1784 | 2107 | 2099 | 1906 | 2340 | 2139 | |
| *Erythrobacteraceae* | 896 | 801 | 689 | 743 | 711 | 652 | 1021 | 1499 | 1071 | 883 | 921 | 1071 | |
| *Sphingomonadaceae* | 7214 | 7565 | 8769 | 7508 | 8297 | 5617 | 5978 | 7600 | 6490 | 5593 | 6242 | 5980 | |
| *Comamonadaceae* | 1152 | 1384 | 746 | 2791 | 2454 | 2371 | 1221 | 1342 | 1118 | 1912 | 1501 | 1174 | |
| *Oxalobacteraceae* | 916 | 940 | 775 | 7161 | 5806 | 5783 | 3417 | 2595 | 2976 | 5912 | 3980 | 2822 | |
| *Polyangiaceae* | 317 | 277 | 282 | 885 | 872 | 1554 | 131 | 184 | 239 | 2647 | 1932 | 1998 | |
| *Enterobacteriaceae* | 2 | 6 | 26 | 429 | 739 | 562 | 1 | 2 | 9 | 831 | 3982 | 1042 | |
| *Sinobacteraceae* | 1363 | 1337 | 1087 | 1180 | 1150 | 1158 | 1078 | 1202 | 1105 | 1205 | 1240 | 1388 | |
| *Xanthomonadaceae* | 4569 | 4397 | 3229 | 7929 | 6308 | 7376 | 8045 | 9351 | 8001 | 8783 | 9106 | 8772 | |
| Others | 14290 | 16870 | 14036 | 13757 | 12901 | 13907 | 14002 | 15810 | 15046 | 14039 | 11603 | 14920 | |
| Unclassified | 45744 | 47022 | 51063 | 33083 | 34280 | 33379 | 36070 | 38372 | 34667 | 28877 | 23310 | 32268 | |
| **Bacterial Family** | **Copy number of 16S rDNA in flowering stage (b) of treatment** | | | | | | | | | | | | |
|  | **bCK1** | **bCK2** | **bCK3** | **bT1** | **bT2** | **bT3** | **bF1** | **bF2** | **bF3** | **bTF1** | **bTF2** | **bTF3** | |
| *Iamiaceae* | 142 | 206 | 128 | 169 | 151 | 111 | 648 | 290 | 188 | 186 | 185 | 324 | |
| *Actinosynnemataceae* | 145 | 473 | 79 | 149 | 47 | 138 | 228 | 253 | 72 | 84 | 239 | 49 | |
| *Micrococcaceae* | 90 | 154 | 70 | 223 | 203 | 165 | 269 | 175 | 72 | 72 | 160 | 74 | |
| *Nocardioidaceae* | 717 | 1078 | 575 | 913 | 894 | 662 | 4350 | 2115 | 861 | 797 | 1743 | 912 | |
| *Streptomycetaceae* | 70 | 214 | 73 | 90 | 63 | 78 | 785 | 319 | 96 | 89 | 312 | 114 | |
| *Gaiellaceae* | 479 | 529 | 721 | 367 | 359 | 249 | 736 | 329 | 187 | 151 | 271 | 175 | |
| *Cytophagaceae* | 1167 | 1566 | 1226 | 2699 | 2338 | 1988 | 4176 | 4171 | 3749 | 3362 | 3425 | 3753 | |
| *Flavobacteriaceae* | 86 | 75 | 195 | 350 | 548 | 362 | 696 | 310 | 665 | 737 | 1114 | 558 | |
| *Sphingobacteriaceae* | 241 | 66 | 131 | 461 | 321 | 207 | 884 | 719 | 541 | 911 | 926 | 753 | |
| *Bacillaceae* | 62 | 188 | 94 | 69 | 79 | 67 | 125 | 72 | 46 | 57 | 162 | 191 | |
| *Pirellulaceae* | 711 | 423 | 305 | 325 | 304 | 649 | 382 | 363 | 270 | 429 | 431 | 163 | |
| *Caulobacteraceae* | 370 | 291 | 300 | 591 | 399 | 394 | 1311 | 1341 | 1306 | 1140 | 1325 | 965 | |
| *Erythrobacteraceae* | 490 | 406 | 430 | 477 | 408 | 287 | 747 | 897 | 1155 | 699 | 1116 | 1271 | |
| *Sphingomonadaceae* | 5736 | 4690 | 5685 | 6338 | 5379 | 4871 | 4538 | 4758 | 5984 | 5508 | 5210 | 6683 | |
| *Comamonadaceae* | 720 | 757 | 713 | 941 | 1367 | 982 | 789 | 590 | 746 | 860 | 764 | 1028 | |
| *Oxalobacteraceae* | 1385 | 481 | 1076 | 2345 | 1714 | 1144 | 1980 | 1982 | 1027 | 1542 | 1350 | 1356 | |
| *Polyangiaceae* | 208 | 231 | 243 | 388 | 349 | 357 | 98 | 71 | 85 | 237 | 278 | 85 | |
| *Enterobacteriaceae* | 5 | 5 | 7 | 298 | 290 | 272 | 19 | 4 | 1 | 734 | 673 | 319 | |
| *Sinobacteraceae* | 761 | 601 | 923 | 984 | 1098 | 1264 | 648 | 611 | 971 | 1032 | 865 | 1179 | |
| *Xanthomonadaceae* | 3055 | 2182 | 2648 | 4687 | 4350 | 3491 | 6410 | 7290 | 6526 | 6528 | 6939 | 7715 | |
| Others | 7908 | 6902 | 7218 | 7476 | 7616 | 6895 | 11184 | 8333 | 7712 | 6399 | 9627 | 6595 | |
| Unclassified | 37029 | 32649 | 35578 | 27513 | 26639 | 27083 | 24438 | 26992 | 23933 | 20663 | 22489 | 29161 | |
| **Bacterial Family** | **Copy number of 16S rDNA in early fruit setting stage (c) of treatment** | | | | | | | | | | | | |
|  | **cCK1** | **cCK2** | **cCK3** | **cT1** | **cT2** | **cT3** | **cF1** | **cF2** | **cF3** | **cTF1** | **cTF2** | **cTF3** | |
| *Iamiaceae* | 230 | 390 | 248 | 321 | 223 | 264 | 523 | 628 | 614 | 470 | 498 | 540 | |
| *Actinosynnemataceae* | 63 | 749 | 187 | 210 | 157 | 139 | 100 | 125 | 265 | 166 | 90 | 69 | |
| *Micrococcaceae* | 165 | 232 | 229 | 561 | 455 | 405 | 183 | 277 | 364 | 127 | 170 | 138 | |
| *Nocardioidaceae* | 1030 | 1723 | 1347 | 1998 | 1382 | 1663 | 1823 | 1502 | 2135 | 1755 | 1407 | 1263 | |
| *Streptomycetaceae* | 163 | 315 | 223 | 180 | 205 | 167 | 197 | 347 | 961 | 232 | 199 | 130 | |
| *Gaiellaceae* | 759 | 1319 | 689 | 915 | 471 | 605 | 339 | 333 | 489 | 299 | 229 | 275 | |
| *Cytophagaceae* | 1120 | 1764 | 1364 | 2018 | 1598 | 1952 | 3284 | 2583 | 2771 | 2754 | 3627 | 3334 | |
| *Flavobacteriaceae* | 32 | 45 | 92 | 59 | 63 | 79 | 900 | 410 | 424 | 476 | 757 | 570 | |
| *Sphingobacteriaceae* | 33 | 127 | 99 | 148 | 158 | 114 | 588 | 315 | 412 | 503 | 600 | 539 | |
| *Bacillaceae* | 153 | 147 | 162 | 221 | 286 | 154 | 165 | 122 | 404 | 192 | 334 | 169 | |
| *Pirellulaceae* | 414 | 978 | 1145 | 207 | 638 | 381 | 804 | 470 | 351 | 266 | 312 | 463 | |
| *Caulobacteraceae* | 311 | 310 | 318 | 398 | 284 | 409 | 1095 | 830 | 880 | 1190 | 898 | 852 | |
| *Erythrobacteraceae* | 469 | 505 | 391 | 636 | 508 | 658 | 1220 | 1168 | 934 | 1334 | 1060 | 901 | |
| *Sphingomonadaceae* | 5219 | 5412 | 3893 | 6700 | 3404 | 5793 | 3715 | 3557 | 3838 | 4423 | 3737 | 3675 | |
| *Comamonadaceae* | 470 | 241 | 574 | 548 | 883 | 1197 | 496 | 287 | 364 | 540 | 743 | 739 | |
| *Oxalobacteraceae* | 355 | 418 | 385 | 1551 | 859 | 1288 | 710 | 312 | 546 | 1074 | 1184 | 1131 | |
| *Polyangiaceae* | 196 | 122 | 140 | 261 | 175 | 283 | 24 | 21 | 27 | 63 | 49 | 54 | |
| *Enterobacteriaceae* | 7 | 5 | 7 | 24 | 47 | 68 | 4 | 8 | 4 | 230 | 537 | 450 | |
| *Sinobacteraceae* | 533 | 458 | 612 | 449 | 450 | 591 | 921 | 695 | 621 | 568 | 900 | 828 | |
| *Xanthomonadaceae* | 2120 | 1611 | 2265 | 3843 | 3666 | 4452 | 5100 | 6168 | 5766 | 5792 | 6790 | 6092 | |
| Others | 7676 | 9526 | 9400 | 8245 | 8457 | 8405 | 8774 | 7110 | 8941 | 6556 | 7943 | 7467 | |
| Unclassified | 36427 | 38002 | 39987 | 31889 | 33144 | 32191 | 28720 | 24441 | 23253 | 21214 | 25383 | 25762 | |
| **Bacterial Family** | **Copy number of 16S rDNA in late fruit setting stage (d) of treatment** | | | | | | | | | | | | |
|  | **dCK1** | **dCK2** | **dCK3** | **dT1** | **dT2** | **dT3** | **dF1** | **dF2** | **dF3** | **dTF1** | **dTF2** | **dTF3** | |
| *Iamiaceae* | 217 | 250 | 237 | 222 | 376 | 303 | 573 | 607 | 554 | 534 | 719 | 937 | |
| *Actinosynnemataceae* | 114 | 103 | 473 | 275 | 1201 | 389 | 107 | 140 | 207 | 230 | 297 | 620 | |
| *Micrococcaceae* | 175 | 243 | 304 | 750 | 671 | 1056 | 322 | 310 | 347 | 820 | 649 | 630 | |
| *Nocardioidaceae* | 1441 | 2174 | 2395 | 1620 | 3577 | 2878 | 3118 | 3104 | 3024 | 2640 | 4393 | 4143 | |
| *Streptomycetaceae* | 119 | 456 | 1040 | 393 | 698 | 783 | 632 | 522 | 543 | 364 | 815 | 871 | |
| *Gaiellaceae* | 741 | 916 | 885 | 595 | 800 | 618 | 576 | 519 | 542 | 412 | 493 | 372 | |
| *Cytophagaceae* | 1076 | 578 | 725 | 1119 | 1667 | 1179 | 948 | 2028 | 1356 | 2658 | 1858 | 2541 | |
| *Flavobacteriaceae* | 74 | 20 | 62 | 36 | 67 | 39 | 171 | 467 | 319 | 597 | 417 | 320 | |
| *Sphingobacteriaceae* | 35 | 26 | 76 | 83 | 213 | 112 | 74 | 150 | 131 | 446 | 227 | 522 | |
| *Bacillaceae* | 208 | 144 | 323 | 247 | 442 | 575 | 316 | 214 | 209 | 993 | 1228 | 491 | |
| *Pirellulaceae* | 387 | 687 | 474 | 218 | 387 | 373 | 322 | 483 | 372 | 232 | 143 | 353 | |
| *Caulobacteraceae* | 183 | 127 | 237 | 179 | 268 | 168 | 366 | 519 | 417 | 492 | 433 | 872 | |
| *Erythrobacteraceae* | 218 | 292 | 405 | 286 | 396 | 297 | 721 | 917 | 810 | 440 | 784 | 438 | |
| *Sphingomonadaceae* | 4946 | 3381 | 3495 | 3185 | 3819 | 2687 | 1773 | 2327 | 2225 | 3115 | 2528 | 2682 | |
| *Comamonadaceae* | 708 | 309 | 1163 | 1241 | 395 | 777 | 604 | 921 | 561 | 1601 | 756 | 235 | |
| *Oxalobacteraceae* | 326 | 182 | 390 | 842 | 869 | 658 | 319 | 504 | 372 | 887 | 480 | 560 | |
| *Polyangiaceae* | 105 | 89 | 106 | 183 | 101 | 125 | 74 | 51 | 49 | 29 | 42 | 14 | |
| *Enterobacteriaceae* | 14 | 4 | 208 | 29 | 81 | 14 | 6 | 3 | 2 | 144 | 140 | 190 | |
| *Sinobacteraceae* | 649 | 248 | 385 | 476 | 355 | 399 | 381 | 713 | 598 | 676 | 436 | 456 | |
| *Xanthomonadaceae* | 2257 | 1084 | 2202 | 3840 | 3000 | 2645 | 2331 | 4525 | 3575 | 6038 | 3918 | 4130 | |
| Others | 7323 | 8024 | 7482 | 6670 | 7773 | 6641 | 7302 | 8686 | 8866 | 7103 | 8429 | 8385 | |
| Unclassified | 32188 | 34773 | 26178 | 24062 | 22168 | 21565 | 23987 | 29122 | 27487 | 21013 | 20456 | 15520 | |
| **Bacterial Family** | **Copy number of 16S rDNA in Original soil** | | | | | |  |  |  |  |  |  |  |
|  | **OS1** | **OS2** | **OS3** |  |  |  |  |  |  |  |  |  |  |
| *Iamiaceae* | 210 | 208 | 240 |  |  |  |  |  |  |  |  |  |  |
| *Actinosynnemataceae* | 333 | 397 | 380 |  |  |  |  |  |  |  |  |  |  |
| *Micrococcaceae* | 136 | 119 | 141 |  |  |  |  |  |  |  |  |  |  |
| *Nocardioidaceae* | 1484 | 1339 | 1592 |  |  |  |  |  |  |  |  |  |  |
| *Streptomycetaceae* | 228 | 199 | 227 |  |  |  |  |  |  |  |  |  |  |
| *Gaiellaceae* | 524 | 594 | 545 |  |  |  |  |  |  |  |  |  |  |
| *Cytophagaceae* | 1807 | 1758 | 1899 |  |  |  |  |  |  |  |  |  |  |
| *Flavobacteriaceae* | 90 | 126 | 87 |  |  |  |  |  |  |  |  |  |  |
| *Sphingobacteriaceae* | 326 | 321 | 362 |  |  |  |  |  |  |  |  |  |  |
| *Bacillaceae* | 51 | 54 | 48 |  |  |  |  |  |  |  |  |  |  |
| *Pirellulaceae* | 329 | 173 | 352 |  |  |  |  |  |  |  |  |  |  |
| *Caulobacteraceae* | 383 | 288 | 365 |  |  |  |  |  |  |  |  |  |  |
| *Erythrobacteraceae* | 322 | 350 | 302 |  |  |  |  |  |  |  |  |  |  |
| *Sphingomonadaceae* | 2979 | 2707 | 2934 |  |  |  |  |  |  |  |  |  |  |
| *Comamonadaceae* | 319 | 238 | 323 |  |  |  |  |  |  |  |  |  |  |
| *Oxalobacteraceae* | 1539 | 1673 | 1627 |  |  |  |  |  |  |  |  |  |  |
| *Polyangiaceae* | 95 | 125 | 132 |  |  |  |  |  |  |  |  |  |  |
| *Enterobacteriaceae* | 8 | 11 | 5 |  |  |  |  |  |  |  |  |  |  |
| *Sinobacteraceae* | 417 | 579 | 479 |  |  |  |  |  |  |  |  |  |  |
| *Xanthomonadaceae* | 1934 | 1759 | 1836 |  |  |  |  |  |  |  |  |  |  |
| Others | 6134 | 6383 | 6467 |  |  |  |  |  |  |  |  |  |  |
| Unclassified | 24675 | 26573 | 27713 |  |  |  |  |  |  |  |  |  |  |

**Table S4**. Abundance of bacteria at the genus level in different growth stages of tomato and different treatments drawn in Figure 2b

| **Bacterial Genus** | **Copy number of 16S rDNA in seedling stage(a) of treatment** | | | | | | | | | | | |  |
| --- | --- | --- | --- | --- | --- | --- | --- | --- | --- | --- | --- | --- | --- |
|  | **aCK1** | **aCK2** | **aCK3** | **aT1** | **aT2** | **aT3** | **aF1** | **aF2** | **aF3** | **aTF1** | **aTF2** | **aTF3** |  |
| *Iamia* | 289 | 187 | 218 | 127 | 96 | 201 | 380 | 216 | 415 | 213 | 185 | 261 |  |
| *Aeromicrobium* | 282 | 332 | 215 | 283 | 213 | 476 | 425 | 228 | 548 | 295 | 477 | 305 |  |
| *Streptomyces* | 291 | 340 | 164 | 400 | 328 | 451 | 597 | 411 | 647 | 537 | 378 | 406 |  |
| *Pontibacter* | 359 | 265 | 292 | 755 | 745 | 783 | 1808 | 1396 | 1727 | 1263 | 1967 | 2083 |  |
| *Flavobacterium* | 440 | 605 | 148 | 2879 | 2147 | 1421 | 635 | 642 | 513 | 3200 | 2427 | 1078 |  |
| *Bacillus* | 171 | 140 | 156 | 101 | 121 | 144 | 146 | 100 | 136 | 128 | 278 | 257 |  |
| *Kaistobacter* | 4083 | 4647 | 5532 | 4421 | 5317 | 3160 | 3871 | 5355 | 3960 | 3985 | 4436 | 4321 |  |
| *Sphingomonasm* | 1367 | 1432 | 1751 | 1775 | 1576 | 1362 | 1012 | 1087 | 1287 | 740 | 860 | 917 |  |
| *Janthinobacterium* | 416 | 469 | 315 | 3098 | 2151 | 2844 | 1247 | 881 | 1085 | 2298 | 1461 | 1209 |  |
| *Sorangium* | 16 | 5 | 14 | 578 | 558 | 1247 | 67 | 111 | 154 | 2591 | 1858 | 1941 |  |
| *Klebsiella* | 0 | 2 | 7 | 266 | 436 | 350 | 0 | 0 | 3 | 487 | 2420 | 633 |  |
| *Arenimonas* | 493 | 499 | 610 | 1060 | 883 | 1049 | 1264 | 1065 | 1131 | 1508 | 1309 | 1449 |  |
| *Thermomonas* | 628 | 561 | 600 | 1352 | 1012 | 1232 | 1961 | 2127 | 2183 | 1622 | 1989 | 1806 |  |
| Others | 10609 | 13638 | 8756 | 20109 | 16671 | 18140 | 14400 | 17281 | 15739 | 18740 | 18583 | 18511 |  |
| Unclassified | 64654 | 66797 | 68100 | 53774 | 53523 | 53190 | 57773 | 60348 | 56916 | 49575 | 41566 | 52364 |  |
| **Bacterial Genus** | **Copy number of 16S rDNA in flowering stage(b) of treatment** | | | | | | | | | | | |  |
|  | **bCK1** | **bCK2** | **bCK3** | **bT1** | **bT2** | **bT3** | **bF1** | **bF2** | **bF3** | **bTF1** | **bTF2** | **bTF3** | |
| *Iamia* | 138 | 199 | 126 | 165 | 149 | 107 | 625 | 279 | 182 | 181 | 183 | 317 | |
| *Aeromicrobium* | 283 | 379 | 212 | 413 | 372 | 225 | 648 | 461 | 333 | 350 | 434 | 335 | |
| *Streptomyces* | 62 | 204 | 64 | 88 | 58 | 75 | 755 | 291 | 90 | 89 | 279 | 111 | |
| *Pontibacter* | 169 | 186 | 184 | 710 | 707 | 465 | 1584 | 1416 | 966 | 1118 | 1044 | 1240 | |
| *Flavobacterium* | 84 | 73 | 194 | 349 | 547 | 362 | 684 | 305 | 664 | 733 | 1110 | 554 | |
| *Bacillus* | 59 | 179 | 85 | 65 | 75 | 62 | 101 | 67 | 42 | 56 | 156 | 187 | |
| *Kaistobacter* | 3341 | 2474 | 3284 | 4073 | 3744 | 3517 | 2839 | 3293 | 4548 | 4382 | 3938 | 5331 | |
| *Sphingomonasm* | 1139 | 1214 | 1275 | 1388 | 904 | 818 | 765 | 759 | 719 | 590 | 629 | 767 | |
| *Janthinobacteriu* | 345 | 190 | 318 | 751 | 492 | 422 | 597 | 645 | 183 | 489 | 312 | 352 | |
| *Sorangium* | 1 | 7 | 0 | 48 | 35 | 20 | 52 | 24 | 54 | 187 | 237 | 20 | |
| *Klebsiella* | 0 | 1 | 0 | 196 | 191 | 172 | 10 | 0 | 0 | 448 | 397 | 173 | |
| *Arenimonas* | 446 | 264 | 427 | 691 | 675 | 655 | 806 | 909 | 954 | 1110 | 663 | 1317 | |
| *Thermomonas* | 607 | 447 | 448 | 954 | 811 | 648 | 1677 | 1968 | 1589 | 1433 | 1489 | 1753 | |
| Others | 5701 | 5071 | 4102 | 7322 | 7279 | 6533 | 12206 | 9371 | 7928 | 8712 | 11552 | 8123 | |
| Unclassified | 49202 | 43279 | 47699 | 40640 | 38877 | 37635 | 42092 | 42197 | 37941 | 32339 | 37181 | 42843 | |
| **Bacterial Genus** | **Copy number of 16S rDNA in early fruit setting stage(c) of treatment** | | | | | | | | | | | |  |
|  | **cCK1** | **cCK2** | **cCK3** | **cT1** | **cT2** | **cT3** | **cF1** | **cF2** | **cF3** | **cTF1** | **cTF2** | **cTF3** | |
| *Iamia* | 219 | 373 | 234 | 304 | 209 | 247 | 488 | 614 | 595 | 449 | 481 | 511 | |
| *Aeromicrobium* | 340 | 363 | 440 | 466 | 371 | 548 | 488 | 313 | 305 | 503 | 337 | 331 | |
| *Streptomyces* | 149 | 294 | 210 | 166 | 194 | 157 | 194 | 336 | 926 | 219 | 190 | 127 | |
| *Pontibacter* | 234 | 310 | 176 | 581 | 431 | 521 | 887 | 887 | 767 | 824 | 1123 | 1166 | |
| *Flavobacterium* | 29 | 43 | 90 | 58 | 63 | 79 | 896 | 405 | 424 | 475 | 747 | 561 | |
| *Bacillus* | 137 | 138 | 158 | 217 | 274 | 154 | 141 | 107 | 319 | 185 | 326 | 156 | |
| *Kaistobacter* | 2418 | 2913 | 1769 | 3304 | 1538 | 3136 | 2506 | 2570 | 2862 | 3156 | 2826 | 2781 | |
| *Sphingomonasm* | 1625 | 1512 | 1148 | 2117 | 1002 | 1518 | 502 | 423 | 416 | 702 | 349 | 392 | |
| *Janthinobacteriu* | 60 | 89 | 69 | 314 | 140 | 205 | 119 | 63 | 97 | 205 | 204 | 200 | |
| *Sorangium* | 9 | 2 | 3 | 7 | 31 | 25 | 4 | 1 | 4 | 12 | 4 | 2 | |
| *Klebsiella* | 0 | 0 | 0 | 10 | 25 | 37 | 0 | 0 | 0 | 139 | 305 | 275 | |
| *Arenimonas* | 195 | 157 | 167 | 285 | 277 | 427 | 694 | 860 | 732 | 744 | 1421 | 903 | |
| *Thermomonas* | 433 | 357 | 468 | 1182 | 768 | 1162 | 1102 | 1453 | 1324 | 1281 | 1396 | 1361 | |
| Others | 4145 | 6309 | 6057 | 6700 | 6925 | 7377 | 8664 | 6852 | 8600 | 8122 | 8499 | 8144 | |
| Unclassified | 47952 | 51539 | 52768 | 45671 | 45265 | 45665 | 43000 | 36825 | 36993 | 33208 | 39239 | 38531 | |
| **Bacterial Genus** | **Copy number of 16S rDNA in late fruit setting stage(d) of treatment** | | | | | | | | | | | |  |
|  | **dCK1** | **dCK2** | **dCK3** | **dT1** | **dT2** | **dT3** | **dF1** | **dF2** | **dF3** | **dTF1** | **dTF2** | **dTF3** | |
| *Iamia* | 204 | 230 | 224 | 215 | 361 | 292 | 553 | 574 | 532 | 509 | 673 | 921 | |
| *Aeromicrobium* | 526 | 775 | 511 | 456 | 992 | 786 | 566 | 714 | 601 | 682 | 879 | 1253 | |
| *Streptomyces* | 109 | 444 | 1012 | 387 | 682 | 759 | 628 | 506 | 524 | 351 | 802 | 825 | |
| *Pontibacter* | 200 | 88 | 111 | 288 | 456 | 334 | 239 | 520 | 334 | 931 | 637 | 961 | |
| *Flavobacterium* | 74 | 20 | 62 | 36 | 66 | 39 | 170 | 465 | 315 | 589 | 408 | 303 | |
| *Bacillus* | 199 | 132 | 310 | 242 | 437 | 570 | 284 | 207 | 197 | 971 | 1208 | 478 | |
| *Kaistobacter* | 2751 | 1227 | 1366 | 1432 | 1873 | 1088 | 1164 | 1392 | 1329 | 2071 | 1711 | 1912 | |
| *Sphingomonasm* | 1119 | 1170 | 1007 | 752 | 1072 | 780 | 303 | 391 | 356 | 314 | 292 | 344 | |
| *Janthinobacteriu* | 40 | 17 | 36 | 123 | 132 | 130 | 36 | 64 | 67 | 124 | 65 | 129 | |
| *Sorangium* | 4 | 3 | 0 | 3 | 3 | 0 | 0 | 0 | 0 | 0 | 1 | 3 | |
| *Klebsiella* | 0 | 0 | 7 | 17 | 44 | 6 | 0 | 0 | 0 | 84 | 78 | 108 | |
| *Arenimonas* | 272 | 44 | 201 | 254 | 257 | 226 | 109 | 399 | 238 | 493 | 227 | 284 | |
| *Thermomonas* | 443 | 277 | 457 | 1041 | 807 | 760 | 632 | 1163 | 931 | 1677 | 967 | 968 | |
| Others | 4606 | 3772 | 6450 | 6352 | 7686 | 5919 | 5798 | 7593 | 7184 | 9394 | 8516 | 10025 | |
| Unclassified | 42957 | 45911 | 37491 | 34953 | 34456 | 32592 | 34541 | 42844 | 39958 | 33274 | 33177 | 26768 | |
| **Bacterial Genus** | **Copy number of 16S rDNA in soil of origin** | | | | | |  |  |  |  |  |  |  |
|  | **OS1** |  | **OS2** |  | **OS3** |  |  |  |  |  |  |  |  |
| *Iamia* | 201 |  | 200 |  | 234 |  |  |  |  |  |  |  |  |
| *Aeromicrobium* | 483 |  | 458 |  | 543 |  |  |  |  |  |  |  |  |
| *Streptomyces* | 183 |  | 158 |  | 175 |  |  |  |  |  |  |  |  |
| *Pontibacter* | 476 |  | 373 |  | 475 |  |  |  |  |  |  |  |  |
| *Flavobacterium* | 86 |  | 107 |  | 77 |  |  |  |  |  |  |  |  |
| *Bacillus* | 47 |  | 48 |  | 45 |  |  |  |  |  |  |  |  |
| *Kaistobacter* | 1246 |  | 989 |  | 1168 |  |  |  |  |  |  |  |  |
| *Sphingomonasm* | 981 |  | 837 |  | 965 |  |  |  |  |  |  |  |  |
| *Janthinobacteriu* | 800 |  | 952 |  | 908 |  |  |  |  |  |  |  |  |
| *Sorangium* | 1 |  | 3 |  | 1 |  |  |  |  |  |  |  |  |
| *Klebsiella* | 3 |  | 2 |  | 0 |  |  |  |  |  |  |  |  |
| *Arenimonas* | 328 |  | 236 |  | 274 |  |  |  |  |  |  |  |  |
| *Thermomonas* | 364 |  | 364 |  | 373 |  |  |  |  |  |  |  |  |
| Others | 5003 |  | 4661 |  | 5067 |  |  |  |  |  |  |  |  |
| Unclassified | 34121 |  | 36586 |  | 37751 |  |  |  |  |  |  |  |  |

**Table S5**. Bacterial taxa with LDA score more than 4 (*P*<0.05) of CK vs T in the seedling stage

| Feature | LDA score | P value | Class_  Hm |
| --- | --- | --- | --- |
| Bacteria.Proteobacteria | 4.84 | 0.049 | aT |
| Bacteria.Bacteroidetes | 4.67 | 0.049 | aT |
| Bacteria.Proteobacteria.Betaproteobacteria.Burkholderiales | 4.62 | 0.049 | aT |
| Bacteria.Proteobacteria.Betaproteobacteria | 4.50 | 0.049 | aT |
| Bacteria.Proteobacteria.Betaproteobacteria.Burkholderiales.Oxalobacteraceae | 4.48 | 0.049 | aT |
| Bacteria.Gemmatimonadetes | 4.48 | 0.049 | aCK |
| Bacteria.Proteobacteria.Gammaproteobacteria | 4.43 | 0.049 | aT |
| Bacteria.Acidobacteria | 4.28 | 0.049 | aCK |
| Bacteria.Proteobacteria.Gammaproteobacteria.Xanthomonadales | 4.25 | 0.049 | aT |
| Bacteria.Proteobacteria.Gammaproteobacteria.Xanthomonadales.Xanthomonadaceae | 4.22 | 0.049 | aT |
| Bacteria.Bacteroidetes.Cytophagia.Cytophagales | 4.21 | 0.049 | aT |
| Bacteria.Bacteroidetes.Cytophagia | 4.16 | 0.049 | aT |
| Bacteria.Acidobacteria.Acidobacteria_6 | 4.14 | 0.049 | aCK |
| Bacteria.Proteobacteria.Betaproteobacteria.Burkholderiales.Oxalobacteraceae.Janthinobacterium | 4.14 | 0.049 | aT |
| Bacteria | 4.11 | 0.049 | aT |
| Bacteria.Gemmatimonadetes.Gemmatimonadetes | 4.09 | 0.049 | aCK |
| Bacteria.Bacteroidetes.Flavobacteriia.Flavobacteriales | 4.09 | 0.049 | aT |
| Bacteria.Bacteroidetes.Cytophagia.Cytophagales.Cytophagaceae | 4.07 | 0.049 | aT |
| Bacteria.Bacteroidetes.Flavobacteriia | 4.06 | 0.049 | aT |
| Bacteria.Acidobacteria.Acidobacteria_6.iii1_15 | 4.02 | 0.049 | aCK |
| Bacteria.Bacteroidetes.Flavobacteriia.Flavobacteriales.Flavobacteriaceae | 4.01 | 0.049 | aT |
| Bacteria.Bacteroidetes.Flavobacteriia.Flavobacteriales.Flavobacteriaceae.Flavobacterium | 4.01 | 0.049 | aT |

**Table S6**. Bacterial taxa with LDA score more than 3.5 (*P*<0.05) of CK vs T in the flowering stage

| Feature | LDA score | P value | Class_  hm |
| --- | --- | --- | --- |
| Bacteria.Proteobacteria | 4.71 | 0.049 | bT |
| Bacteria.Bacteroidetes | 4.41 | 0.049 | bT |
| Bacteria.Proteobacteria.Gammaproteobacteria | 4.38 | 0.049 | bT |
| Bacteria.Proteobacteria.Gammaproteobacteria.Xanthomonadales | 4.29 | 0.049 | bT |
| Bacteria.Acidobacteria | 4.23 | 0.049 | bCK |
| Bacteria.Actinobacteria | 4.23 | 0.049 | bCK |
| Bacteria.Proteobacteria.Gammaproteobacteria.Xanthomonadales.Xanthomonadaceae | 4.17 | 0.049 | bT |
| Bacteria.Acidobacteria.Acidobacteria_6.iii1_15 | 4.13 | 0.049 | bCK |
| Bacteria.Acidobacteria.Acidobacteria_6 | 4.13 | 0.049 | bCK |
| Bacteria.Proteobacteria.Betaproteobacteria.Burkholderiales | 4.07 | 0.049 | bT |
| Bacteria.Bacteroidetes.Cytophagia.Cytophagales | 4.07 | 0.049 | bT |
| Bacteria.Bacteroidetes.Cytophagia | 4.05 | 0.049 | bT |
| Bacteria.Proteobacteria.Alphaproteobacteria.Sphingomonadales.Sphingomonadaceae.Kaistobacter | 4.03 | 0.049 | bT |
| Bacteria.Actinobacteria.Acidimicrobiia | 4.02 | 0.049 | bCK |
| Bacteria.Actinobacteria.Acidimicrobiia.Acidimicrobiales | 3.91 | 0.049 | bCK |
| Bacteria.Bacteroidetes.Cytophagia.Cytophagales.Cytophagaceae | 3.91 | 0.049 | bT |
| Bacteria.Bacteroidetes.Bacteroidia.Bacteroidales | 3.82 | 0.049 | bT |
| Bacteria.Bacteroidetes.Bacteroidia | 3.75 | 0.049 | bT |
| Bacteria.Gemmatimonadetes.Gemm_5 | 3.74 | 0.049 | bCK |
| Bacteria.Actinobacteria.Thermoleophilia | 3.67 | 0.049 | bCK |
| Bacteria.Proteobacteria.Deltaproteobacteria.Myxococcales | 3.65 | 0.049 | bT |
| Bacteria.Bacteroidetes.Cytophagia.Cytophagales.Cytophagaceae.Pontibacter | 3.64 | 0.049 | bT |
| Bacteria.Proteobacteria.Gammaproteobacteria.Xanthomonadales.Sinobacteraceae | 3.63 | 0.049 | bT |
| Bacteria.Proteobacteria.Gammaproteobacteria.Xanthomonadales.Xanthomonadaceae.Lysobacter | 3.55 | 0.049 | bT |
| Bacteria.Bacteroidetes.Flavobacteriia | 3.52 | 0.049 | bT |
| Bacteria.Proteobacteria.Betaproteobacteria.Burkholderiales.Comamonadaceae | 3.51 | 0.049 | bT |
| Bacteria.Bacteroidetes.Flavobacteriia.Flavobacteriales | 3.51 | 0.049 | bT |

**Table S7**. Bacterial taxa with LDA score more than 3.5 (*P*<0.05) of CK vs T

in early fruit setting stage

| Feature | LDA score | p value | Class_hm |
| --- | --- | --- | --- |
| Bacteria.Proteobacteria.Gammaproteobacteria | 4.34 | 0.049 | cT |
| Bacteria.Proteobacteria.Gammaproteobacteria.Xanthomonadales | 4.26 | 0.049 | cT |
| Bacteria.Proteobacteria.Gammaproteobacteria.Xanthomonadales.Xanthomonadaceae | 4.24 | 0.049 | cT |
| Bacteria.Bacteroidetes | 4.16 | 0.049 | cT |
| Bacteria.Proteobacteria.Betaproteobacteria.Burkholderiales | 4.10 | 0.049 | cT |
| Bacteria.Acidobacteria | 4.03 | 0.049 | cCK |
| Bacteria.Proteobacteria.Betaproteobacteria | 4.00 | 0.049 | cT |
| Bacteria.Bacteroidetes.Bacteroidia.Bacteroidales | 3.94 | 0.049 | cT |
| Bacteria.Bacteroidetes.Bacteroidia | 3.89 | 0.049 | cT |
| Bacteria.Proteobacteria.Betaproteobacteria.Burkholderiales.Oxalobacteraceae | 3.81 | 0.049 | cT |
| Bacteria.Proteobacteria.Gammaproteobacteria.Xanthomonadales.Xanthomonadaceae.Thermomonas | 3.77 | 0.049 | cT |
| Bacteria.Bacteroidetes.Cytophagia | 3.71 | 0.049 | cT |
| Bacteria.Bacteroidetes.Cytophagia.Cytophagales | 3.56 | 0.049 | cT |

**Table S8**. Bacterial taxa with LDA score more than 3.5 (*P*<0.05) of CK vs T

in late fruit setting stage

| Feature | LDA score | P value | Class_  hm |
| --- | --- | --- | --- |
| Bacteria.Proteobacteria.Gammaproteobacteria.Xanthomonadales | 4.22 | 0.049 | dT |
| Bacteria.Proteobacteria.Gammaproteobacteria.Xanthomonadales.Xanthomonadaceae | 4.21 | 0.049 | dT |
| Bacteria.Acidobacteria | 4.17 | 0.049 | dCK |
| Bacteria.Proteobacteria.Gammaproteobacteria | 4.15 | 0.049 | dT |
| Bacteria.Acidobacteria.Acidobacteria_6.iii1_15 | 4.03 | 0.049 | dCK |
| Bacteria.Acidobacteria.Acidobacteria_6 | 4.03 | 0.049 | dCK |
| Bacteria.Bacteroidetes | 3.98 | 0.049 | dT |
| Bacteria.Planctomycetes | 3.98 | 0.049 | dCK |
| Bacteria.Planctomycetes.Phycisphaerae | 3.86 | 0.049 | dCK |
| Bacteria.Planctomycetes.Phycisphaerae.WD2101 | 3.86 | 0.049 | dCK |
| Bacteria.Bacteroidetes.Cytophagia.Cytophagales | 3.85 | 0.049 | dT |
| Bacteria.Bacteroidetes.Cytophagia.Cytophagales.Cytophagaceae | 3.82 | 0.049 | dT |
| Bacteria.Actinobacteria.Actinobacteria.Actinomycetales.Micrococcaceae | 3.82 | 0.049 | dT |
| Bacteria.Proteobacteria.Gammaproteobacteria.Xanthomonadales.Xanthomonadaceae.Thermomonas | 3.77 | 0.049 | dT |
| Bacteria.Bacteroidetes.Cytophagia | 3.76 | 0.049 | dT |
| Bacteria.Proteobacteria.Betaproteobacteria.Burkholderiales.Oxalobacteraceae | 3.75 | 0.049 | dT |
| Bacteria.Proteobacteria.Gammaproteobacteria.Xanthomonadales.Xanthomonadaceae.Lysobacter | 3.57 | 0.049 | dT |

**Table S9**. Bacterial taxa with LDA score more than 4 (*P*<0.05) of F vs TF in seedling stage

| Feature | LDA score | P value | Class_  hm |
| --- | --- | --- | --- |
| Bacteria.Proteobacteria | 4.56 | 0.049 | aTF |
| Bacteria.Bacteroidetes | 4.40 | 0.049 | aTF |
| Bacteria.Actinobacteria | 4.34 | 0.049 | aF |
| Bacteria.Proteobacteria.Gammaproteobacteria | 4.23 | 0.049 | aTF |
| Bacteria.Proteobacteria.Deltaproteobacteria.Myxococcales | 4.16 | 0.049 | aTF |
| Bacteria.Proteobacteria.Deltaproteobacteria | 4.14 | 0.049 | aTF |
| Bacteria.Bacteroidetes.Flavobacteriia.Flavobacteriales | 4.10 | 0.049 | aTF |
| Bacteria.Proteobacteria.Deltaproteobacteria.Myxococcales.Polyangiaceae.Sorangium | 4.08 | 0.049 | aTF |
| Bacteria.Proteobacteria.Gammaproteobacteria.Enterobacteriales.Enterobacteriaceae | 4.08 | 0.049 | aTF |
| Bacteria.Bacteroidetes.Flavobacteriia | 4.07 | 0.049 | aTF |
| Bacteria.Proteobacteria.Deltaproteobacteria.Myxococcales.Polyangiaceae.Sorangium.cellulosum | 4.07 | 0.049 | aTF |
| Bacteria.Proteobacteria.Deltaproteobacteria.Myxococcales.Polyangiaceae | 4.07 | 0.049 | aTF |
| Bacteria.Actinobacteria.Actinobacteria.Actinomycetales | 4.06 | 0.049 | aF |
| Bacteria.Actinobacteria.Actinobacteria | 4.05 | 0.049 | aF |
| Bacteria.Proteobacteria.Gammaproteobacteria.Enterobacteriales | 4.05 | 0.049 | aTF |
| Bacteria.Bacteroidetes.Sphingobacteriia | 4.02 | 0.049 | aTF |
| Bacteria.Bacteroidetes.Sphingobacteriia.Sphingobacteriales | 4.01 | 0.049 | aTF |
| Bacteria.Bacteroidetes.Flavobacteriia.Flavobacteriales.Flavobacteriaceae | 4.00 | 0.049 | aTF |

**Table S10**. Bacterial taxa with LDA score more than 3.3 (*P*<0.05) of F vs TF in flowering stage

| Feature | LDA score | P value | Class_  hm |
| --- | --- | --- | --- |
| Bacteria.Bacteroidetes | 4.23 | 0.049 | bTF |
| Bacteria.Actinobacteria.Acidimicrobiia | 4.04 | 0.049 | bF |
| Bacteria.Actinobacteria.Acidimicrobiia.Acidimicrobiales | 4.00 | 0.049 | bF |
| Bacteria.Bacteroidetes.Bacteroidia | 3.98 | 0.049 | bTF |
| Bacteria.Bacteroidetes.Bacteroidia.Bacteroidales | 3.92 | 0.049 | bTF |
| Bacteria.Proteobacteria.Gammaproteobacteria.Enterobacteriales.Enterobacteriaceae | 3.69 | 0.049 | bTF |
| Bacteria.Proteobacteria.Gammaproteobacteria.Enterobacteriales | 3.67 | 0.049 | bTF |
| Bacteria.Bacteroidetes.Sphingobacteriia | 3.48 | 0.049 | bTF |
| Bacteria.Proteobacteria.Gammaproteobacteria.Enterobacteriales.Enterobacteriaceae.Klebsiella | 3.47 | 0.046 | bTF |
| Bacteria.Bacteroidetes.Sphingobacteriia.Sphingobacteriales | 3.47 | 0.049 | bTF |
| Bacteria.Proteobacteria.Deltaproteobacteria.Myxococcales | 3.39 | 0.049 | bTF |
| Bacteria.Actinobacteria.Actinobacteria.Actinomycetales.Glycomycetaceae | 3.35 | 0.049 | bF |
| Bacteria.Actinobacteria.Actinobacteria.Actinomycetales.Glycomycetaceae.Glycomyces | 3.34 | 0.049 | bF |
| Bacteria.Actinobacteria.Actinobacteria.Actinomycetales.Glycomycetaceae.Glycomyces.harbinensis | 3.31 | 0.049 | bF |

**Table S11**. Bacterial taxa with LDA score more than 3.3 (*P*<0.05) of F vs TF

in early fruit setting stage

| Feature | LDA score | P value | Class_  hm | |
| --- | --- | --- | --- | --- |
| Bacteria.Proteobacteria.Betaproteobacteria | 4.14 | 0.049 | cTF | |
| Bacteria.Proteobacteria | 4.06 | 0.049 | cTF | |
| Bacteria.Bacteroidetes | 4.02 | 0.049 | cTF |  |
| Bacteria.Proteobacteria.Betaproteobacteria.Burkholderiales | 3.96 | 0.049 | cTF | |
| Bacteria.Actinobacteria.Acidimicrobiia | 3.78 | 0.049 | cF | |
| Bacteria.Proteobacteria.Betaproteobacteria.Burkholderiales.Oxalobacteraceae | 3.76 | 0.049 | cTF | |
| Bacteria.Bacteroidetes.Cytophagia.Cytophagales | 3.75 | 0.049 | cTF | |
| Bacteria.Actinobacteria.Acidimicrobiia.Acidimicrobiales | 3.71 | 0.049 | cF | |
| Bacteria.TM7.TM7_3 | 3.66 | 0.049 | cF | |
| Bacteria.TM7 | 3.61 | 0.049 | cF | |
| Bacteria.Proteobacteria.Gammaproteobacteria.Enterobacteriales.Enterobacteriaceae | 3.58 | 0.049 | cTF | |
| Bacteria.Proteobacteria.Gammaproteobacteria.Enterobacteriales | 3.58 | 0.049 | cTF | |
| Bacteria.Chloroflexi | 3.56 | 0.049 | cF | |
| Bacteria.Bacteroidetes.Bacteroidia | 3.56 | 0.049 | cTF | |
| Bacteria.Bacteroidetes.Cytophagia | 3.51 | 0.049 | cTF | |
| Bacteria.Proteobacteria.Betaproteobacteria.Burkholderiales.Comamonadaceae | 3.46 | 0.049 | cTF | |
| Bacteria.Proteobacteria.Deltaproteobacteria | 3.40 | 0.049 | cTF | |
| Bacteria.Proteobacteria.Gammaproteobacteria.Enterobacteriales.Enterobacteriaceae.Klebsiella | 3.37 | 0.036 | cTF | |
| Bacteria.Actinobacteria.MB_A2_108 | 3.33 | 0.049 | cF | |
| Bacteria.Bacteroidetes.Bacteroidia.Bacteroidales | 3.33 | 0.049 | cTF | |

**Table S12**. Bacterial taxa with LDA score more than 3.5 (*P*<0.05) of F vs TF

in late fruit setting stage

| Feature | LDA score | p value | Class_  hm |  |
| --- | --- | --- | --- | --- |
| Bacteria.Acidobacteria | 4.30 | 0.049 | dF |  |
| Bacteria.Acidobacteria.Acidobacteria_6.iii1_15 | 4.15 | 0.049 | dF |  |
| Bacteria.Acidobacteria.Acidobacteria_6 | 4.14 | 0.049 | dF | |
| Bacteria.Bacteroidetes | 4.13 | 0.049 | dTF |  |
| Bacteria.Planctomycetes | 4.06 | 0.049 | dF |  |
| Bacteria.Gemmatimonadetes.Gemmatimonadetes | 4.00 | 0.049 | dF |  |
| Bacteria.Bacteroidetes.Cytophagia | 4.00 | 0.049 | dTF |  |
| Bacteria.Bacteroidetes.Cytophagia.Cytophagales.Cytophagaceae | 3.96 | 0.049 | dTF |  |
| Bacteria.Bacteroidetes.Cytophagia.Cytophagales | 3.94 | 0.049 | dTF |  |
| Bacteria.Firmicutes.Bacilli.Bacillales | 3.88 | 0.049 | dTF |  |
| Bacteria.Firmicutes.Bacilli.Bacillales.Bacillaceae.Bacillus | 3.88 | 0.049 | dTF |  |
| Bacteria.Planctomycetes.Phycisphaerae | 3.86 | 0.049 | dF |  |
| Bacteria.Firmicutes.Bacilli.Bacillales.Bacillaceae | 3.85 | 0.049 | dTF |  |
| Bacteria.Firmicutes | 3.85 | 0.049 | dTF |  |
| Bacteria.Firmicutes.Bacilli | 3.85 | 0.049 | dTF |  |
| Bacteria.Proteobacteria.Alphaproteobacteria.Sphingomonadales.Sphingomonadaceae | 3.84 | 0.049 | dTF |  |
| Bacteria.Planctomycetes.Phycisphaerae.WD2101 | 3.80 | 0.049 | dF |  |
| Bacteria.Proteobacteria.Alphaproteobacteria.Sphingomonadales.Sphingomonadaceae.Kaistobacter | 3.79 | 0.049 | dTF |  |
| Bacteria.Firmicutes.Bacilli.Bacillales.Bacillaceae.Bacillus.fumarioli | 3.73 | 0.049 | dTF |  |
| Bacteria.Bacteroidetes.Cytophagia.Cytophagales.Cytophagaceae.Pontibacter | 3.72 | 0.049 | dTF |  |
| Bacteria.Proteobacteria.Alphaproteobacteria | 3.72 | 0.049 | dTF |  |
| Bacteria.Proteobacteria.Betaproteobacteria.Ellin6067 | 3.67 | 0.049 | dF |  |
| Bacteria.Proteobacteria.Deltaproteobacteria | 3.67 | 0.049 | dF |  |
| Bacteria.Proteobacteria.Alphaproteobacteria.Sphingomonadales | 3.63 | 0.049 | dTF |  |
| Bacteria.Actinobacteria.Actinobacteria.Actinomycetales.Micrococcaceae | 3.60 | 0.049 | dTF |  |
| Bacteria.Actinobacteria.Actinobacteria.Actinomycetales.Nocardioidaceae.Aeromicrobium | 3.51 | 0.049 | dTF |  |

**Table S13**. Growth promoting effect of isolated strains from rhizosphere or endosphere

| strains | Aseptic germination experiment in room | | Germination experiment in greenhouse | |
| --- | --- | --- | --- | --- |
|  | Ratio of germination(%) | Length of bud(cm) | Ratio of germination(%) | Plant dry weight(g) |
| CK | 90 | 9.13 | 56.48 | 1.72 |
| WSW001 | 96.665 | 9.33 | 67.59 | 2.45 |
| WSW002 | 96.665 | 9.92 | 76.84 | 2.89 |
| WSW003 | 100 | 9.18 | 58.79 | 2.39 |
| WSW004 | 93.33 | 9.67 | 57.86 | 2.11 |
| WSW007 | 100 | 9.78 | 71.29 | 2.37 |
| TOR3209 | 96.665 | 9.35 | 68.51 | 3.07 |


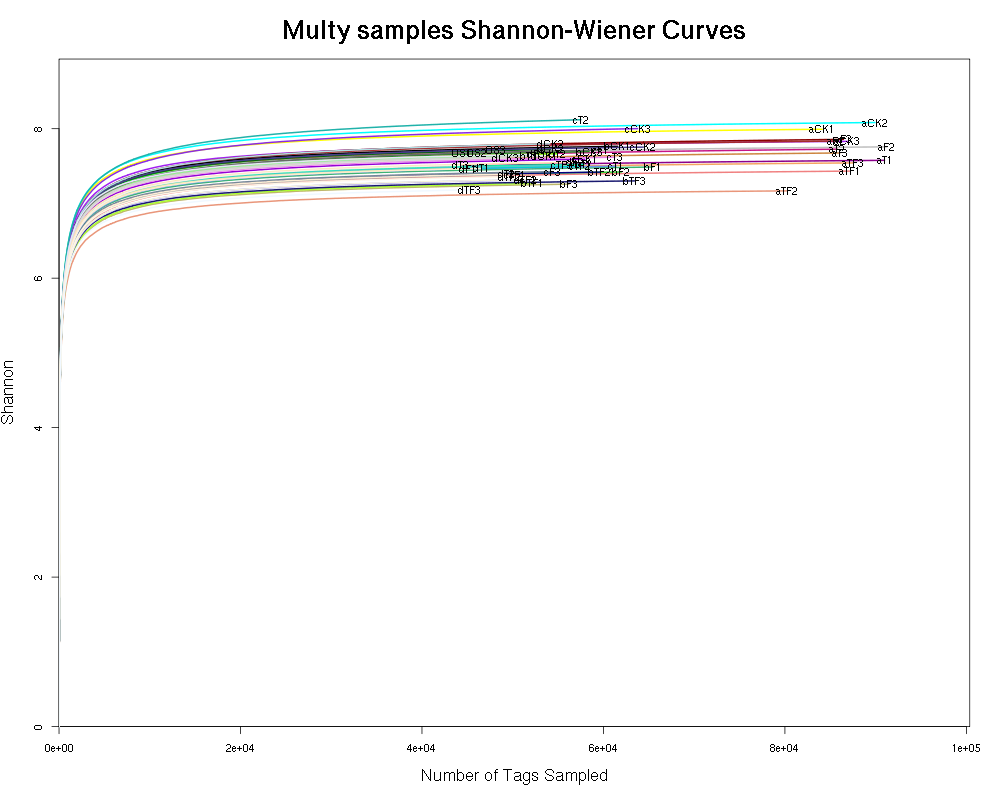


**(a)**


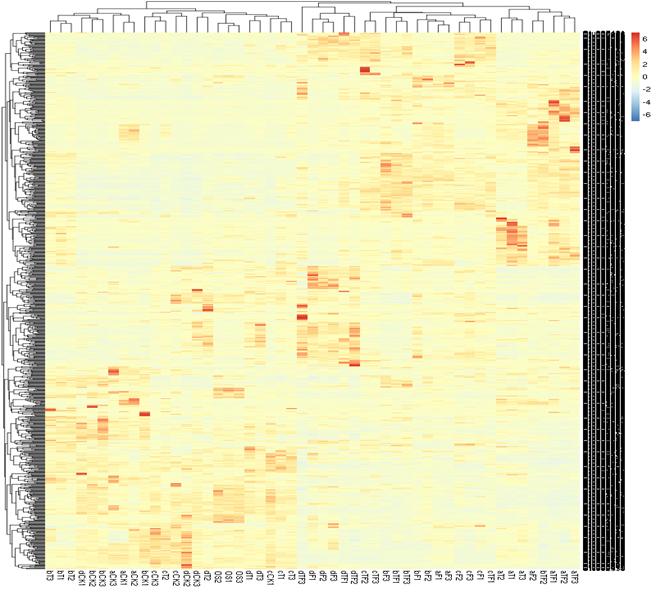


**(b)**

No fertilizer soil Fertilizer soil

**
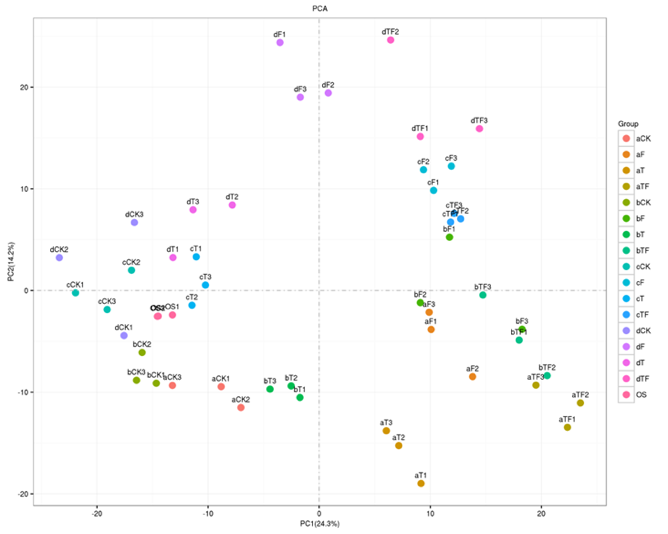
**

**(c)**

**
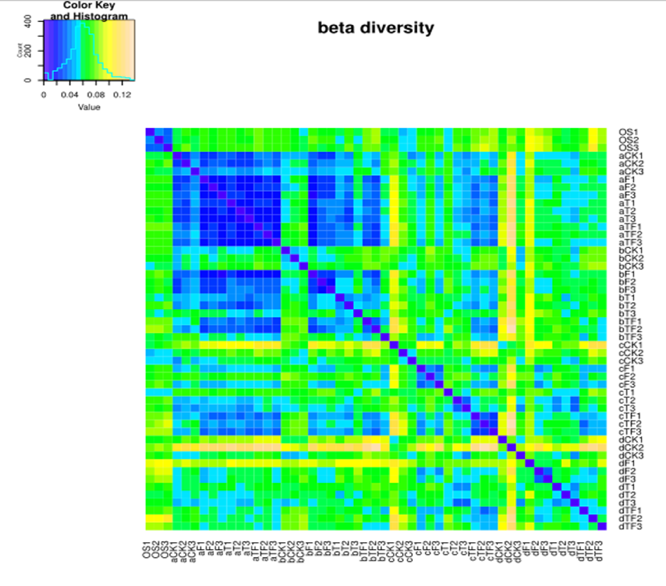
**

**(d)**

**Supplementary Fig. S1.** Comparison of microbial community structure in rhizosphere soil of the four treatments. **(a)** The Shannon rarefaction curve indicating sufficient sequencing capacity (Mothur v.1.34.0, https://mothur.org/). The abscissa represented the number of tags extracted; the ordinate represented the expected value of Shannon calculated when a certain number of tags are extracted; **(b)** The heatmap reflecting OTU expression of all samples; **(c)** The PCA plot reflecting the relationship between samples; **(d)** The Beta diversity heatmap reflecting differences in species diversity between different samples. The pheatmap (v. 3.2.1) and gmodels (v. 2.16.2) package in R were used to make figures (b), (c), and (d) (https://cran.r-project.org/).

**(a)**


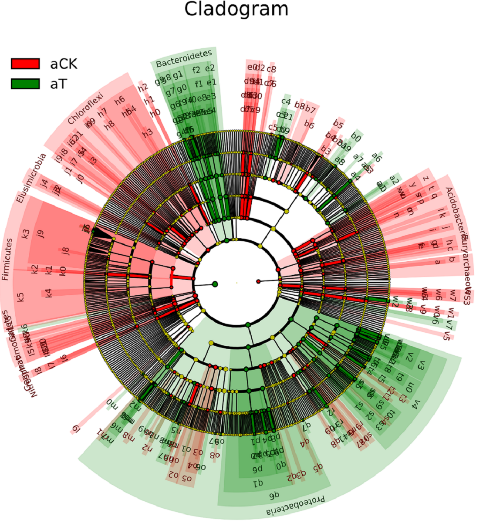

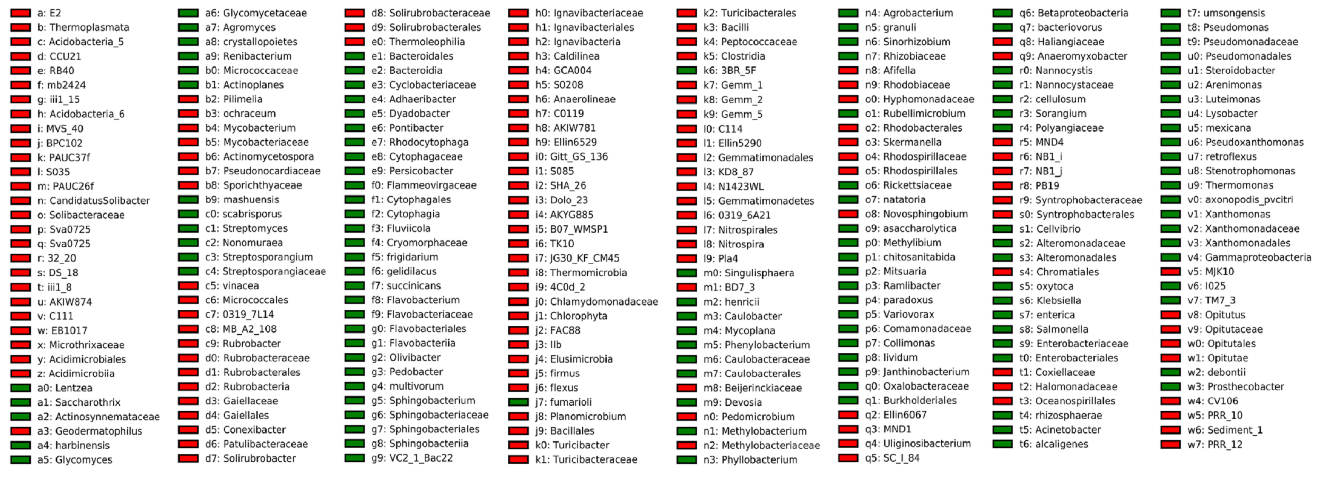


**(b)**


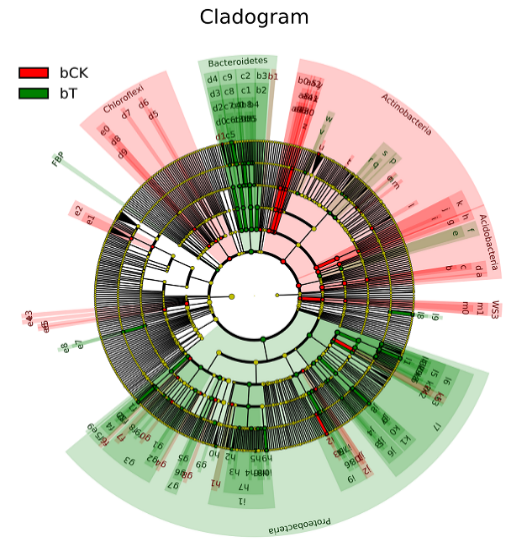

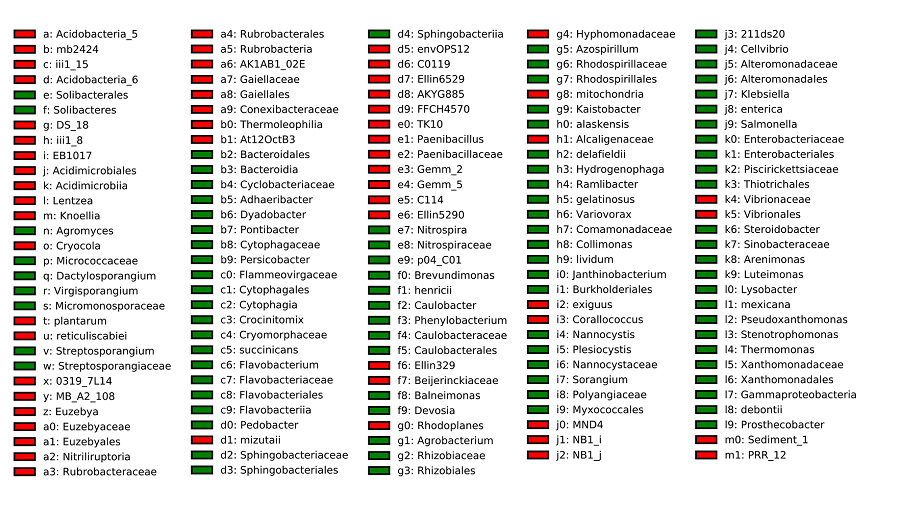


**(c)**


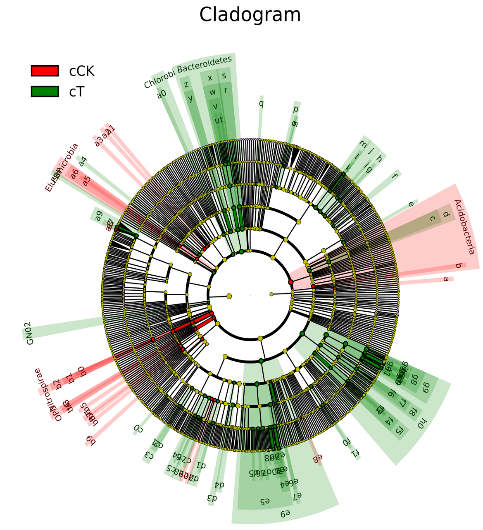

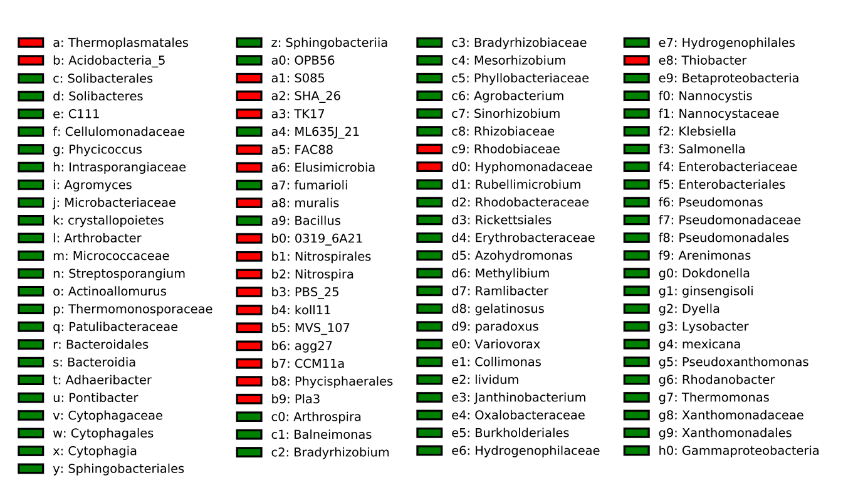


**Supplementary Fig.  S2.** Cladogram plotted from LefSe (https://huttenhower.sph.harvard.edu/galaxy/) comparison analysis indicating the taxonomic representation of statistically and biologically consistent differences of identified biomarkers among group CK and treatment T. The taxonomic levels are represented by rings: the outermost ring = phyla, the innermost ring = genera. Each dot in the ring corresponds a taxon at that level. The taxa at each level are colored according to the treatment: taxa without significant differences are uniformly colored yellow. The taxa in green color were those more abundant in the treatment T than in CK, while those in red color were more abundant in CK than in T. (a) seedling stage; (b) flowering stage; (c) early fruit setting stage; (d) late fruit setting stage respectively.

**(d)**


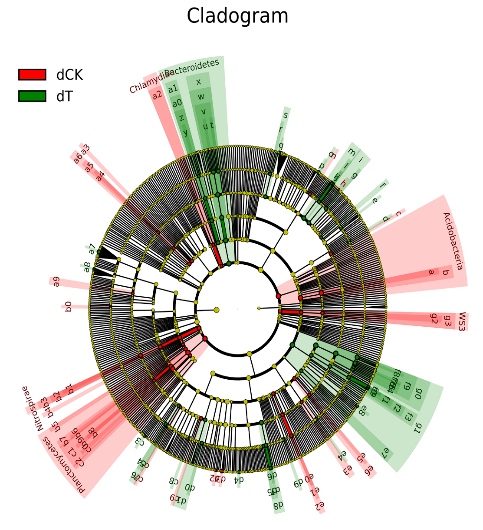

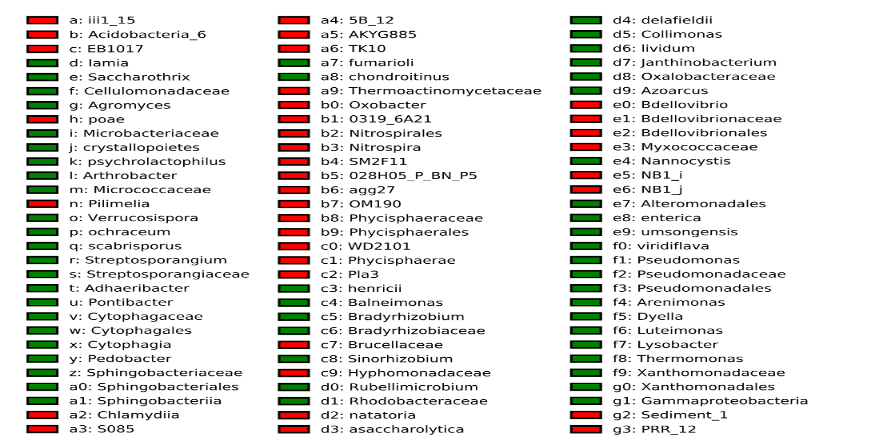

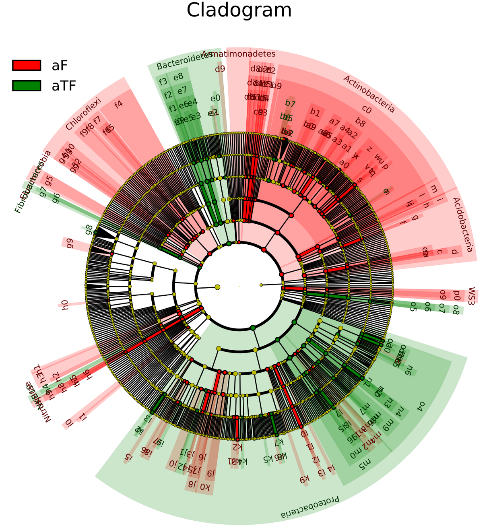

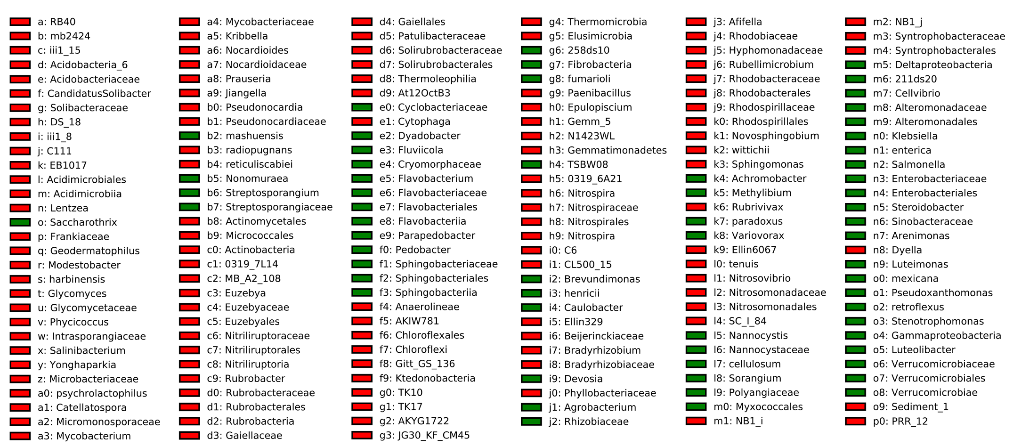


**(a)**

**(c)**


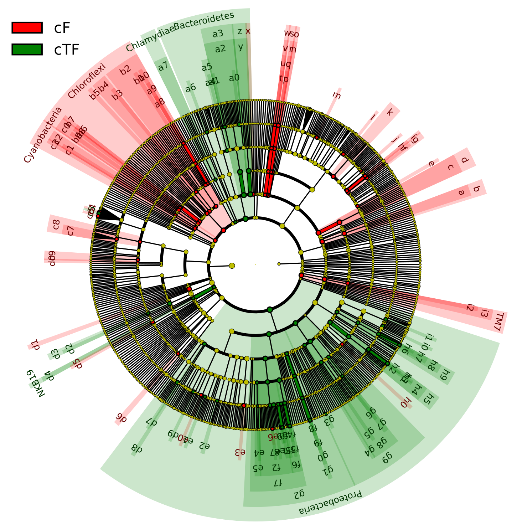

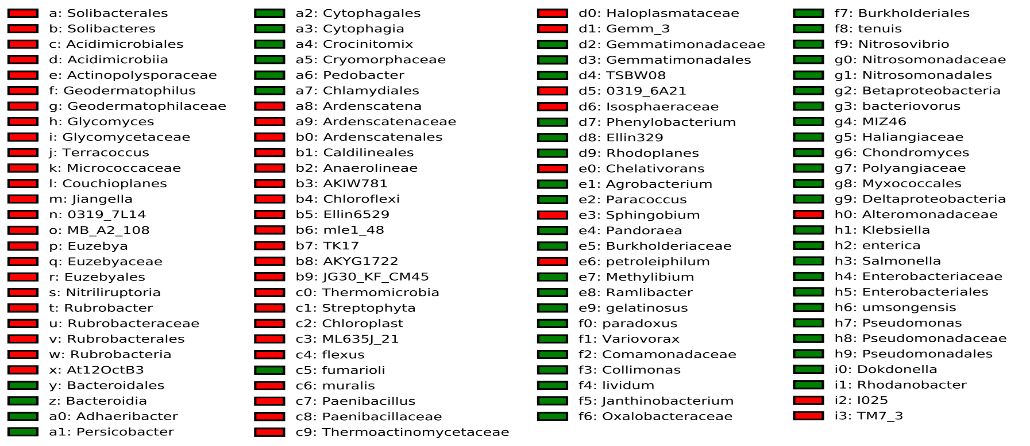

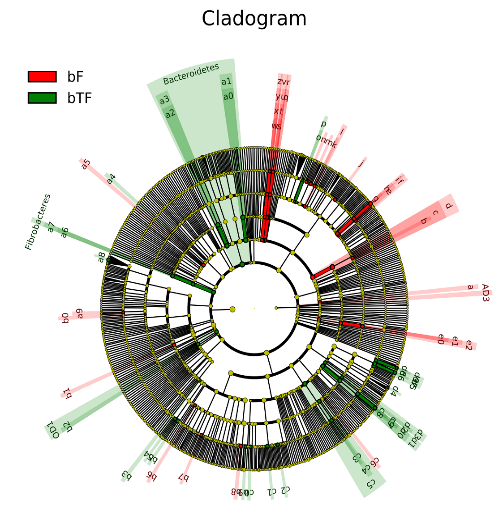

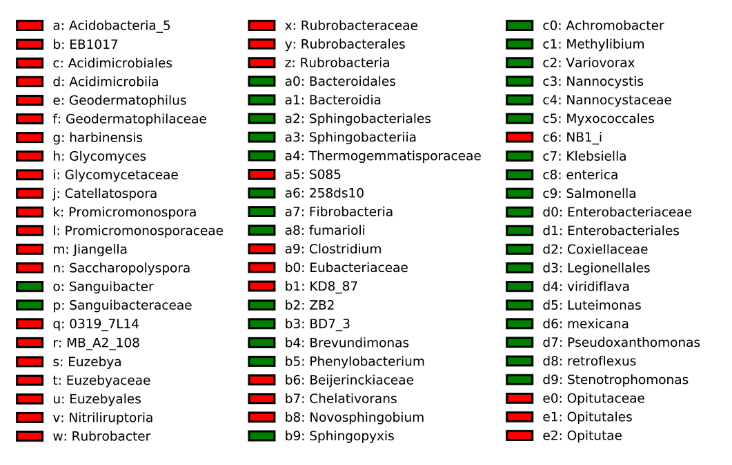


**(b)**

(d)


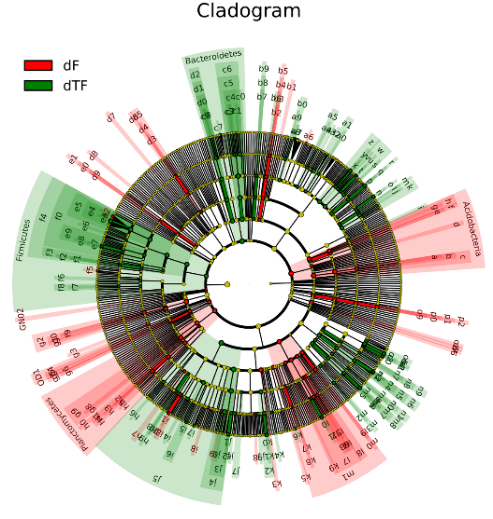

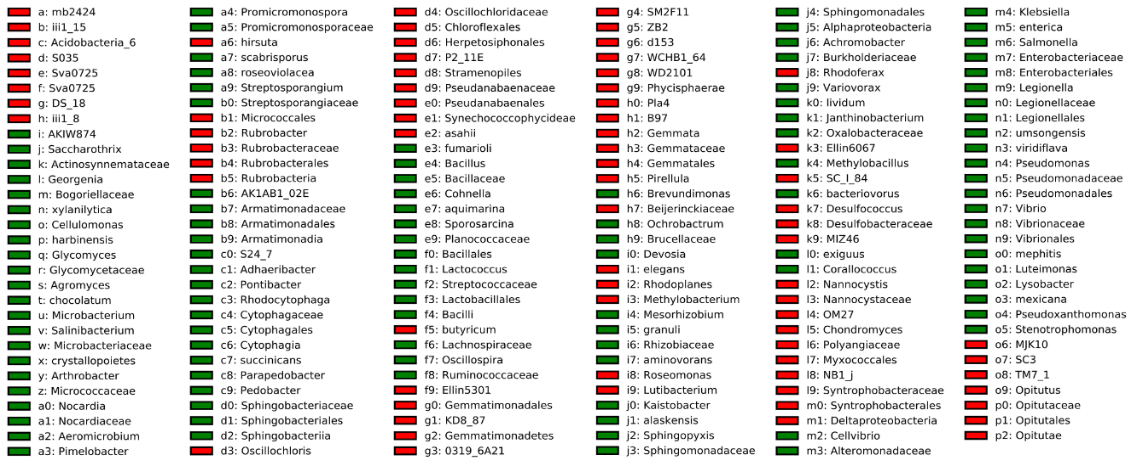


**Supplementary Fig. S3.** Cladogram plotted from LEfSe (https://huttenhower.sph.harvard.edu/galaxy/) comparison analysis indicating the taxonomic representation of statistically and biologically consistent differences of identified biomarkers among group F and TF. The taxonomic levels are represented by rings: the outermost ring = phyla, the innermost ring = genera. Each dot in the ring corresponds a taxon at that level. The taxa at each level are colored according to the treatment: taxa without significant differences are uniformly colored yellow. The taxa in green color were those more abundant in the treatment TF than in F, while those in red color were more abundant in F than in TF. (a) seedling stage; (b) flowering stage; (c) early fruit setting stage; (d) late fruit setting stage respectively.

**
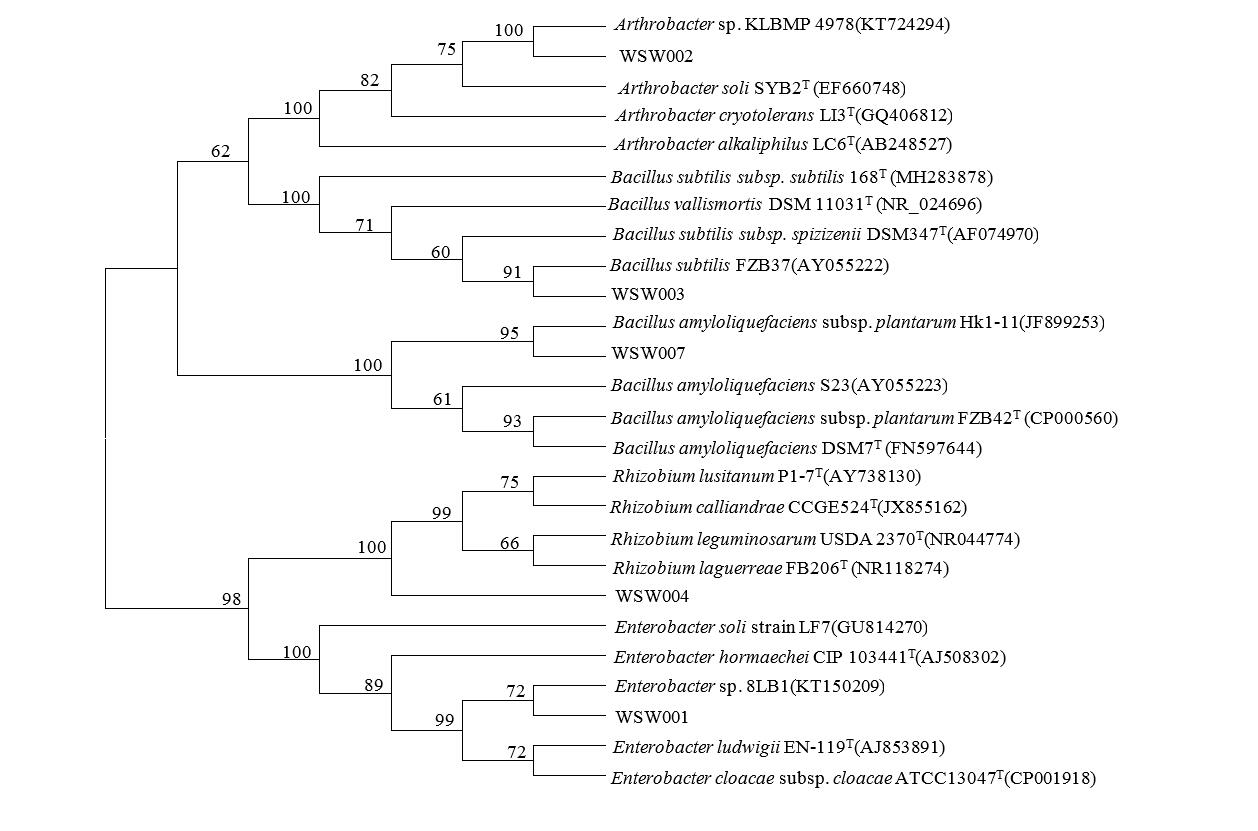
**

**Supplementary Fig. S4.** Phylogenetic tree based on 16S rDNA sequence of the isolates reflecting their taxonomic affiliation. The tree was constructed by Neighbor-joining method.


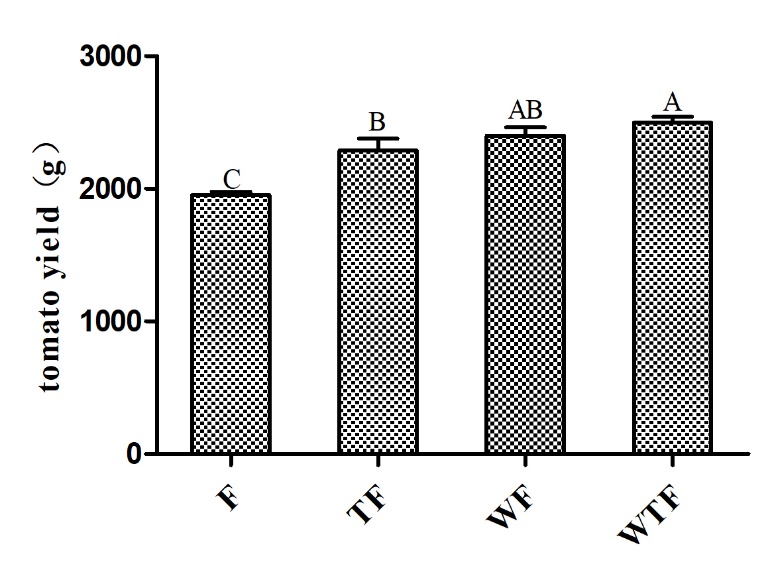

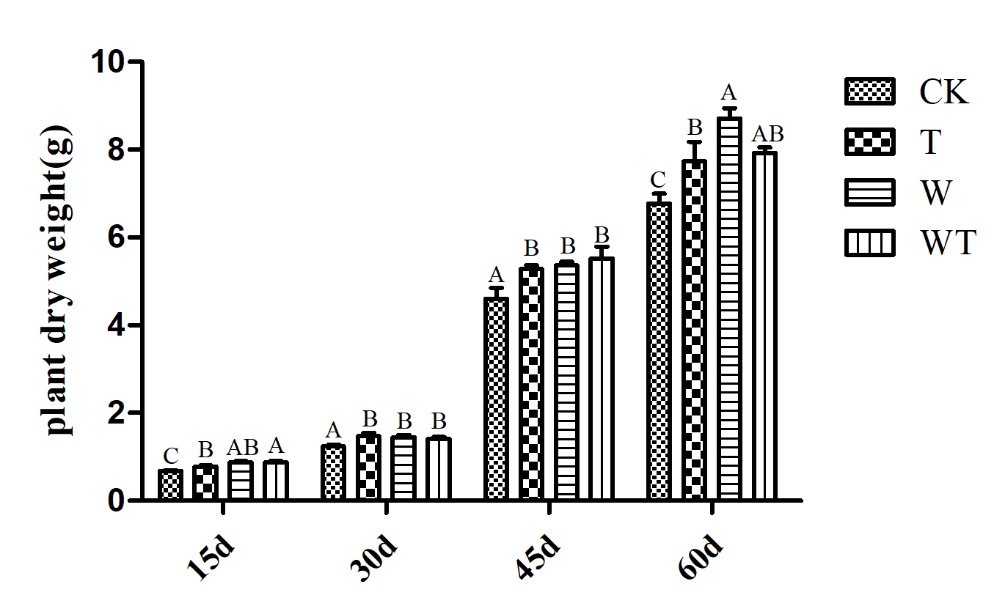
**Supplementary Fig. S5.** The plant dry weight every 15 days of different treatments. (CK: natural loam soil; T: natural loam soil supplied with TOR3209 at the dose of 10^7^ CFU/g; W: natural loam soil supplied with WSW007 at the dose of 10^5^ CFU/g; WT: group T supplied with 10^5^ CFU/g WSW007)

**Supplementary Fig. S6.** Total yield (four plants) from four different treatment groups. (F: natural loam soil supplied with 10% (w/w) organic fertilizer; TF: group F supplied with TOR3209 at the dose of 10^7^ CFU/g; WF: group F supplied with WSW007 at the dose of 10^5^ CFU/g; WTF: group TF supplied with 10^5^ CFU/g WSW007)


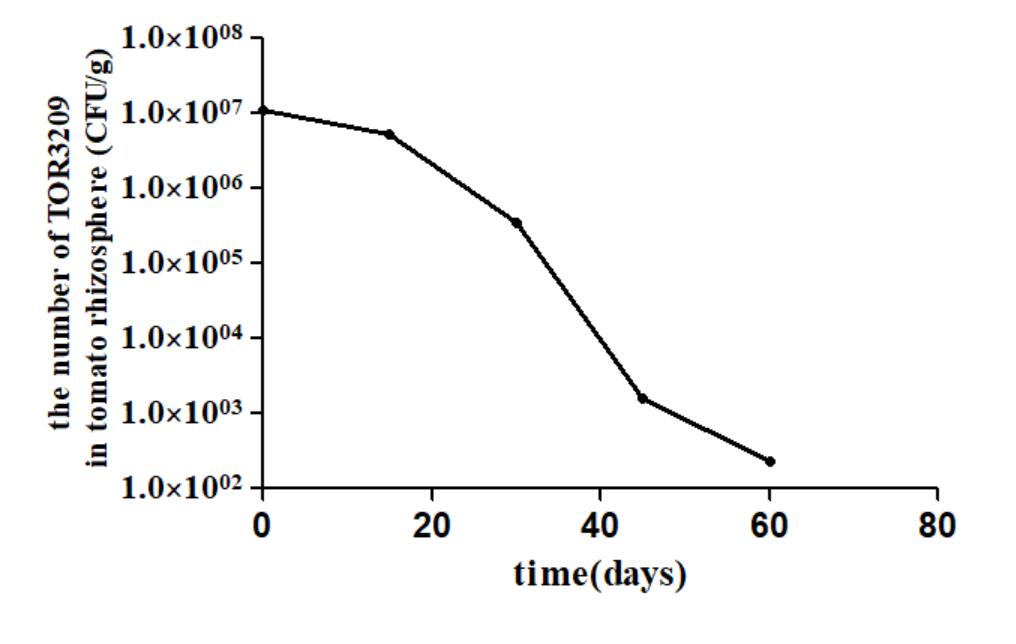


**Supplementary Fig. S7.** The number of TOR3209 in tomato rhizosphere soil over time
